# Psychological impact and resilience among healthcare workers following the 2023 Kahramanmaraş earthquakes: systematic review and meta-analysis

**DOI:** 10.3389/fpsyg.2026.1779042

**Published:** 2026-10-07

**Authors:** Metin Çınaroğlu, Eda Yılmazer

**Affiliations:** 1 Department of Psychology, Faculty of Administrative and Social Science, İstanbul Nişantaşı University, İstanbul, Türkiye; 2 Department of Psychology, Faculty of Arts and Sciences, Haliç University, İstanbul, Türkiye

**Keywords:** coping strategies, earthquake, healthcare workers, meta analysis, post-traumatic stress disorder, resilience

## Abstract

**Background:**

Healthcare workers are uniquely vulnerable to psychological distress following large-scale disasters, through simultaneous exposure as survivors and responders. The 2023 Kahramanmaraş earthquakes placed extraordinary demands on frontline health professionals in Türkiye. This mixed-methods systematic review and meta-analysis aimed to synthesise quantitative and qualitative evidence on post-traumatic psychological outcomes and resilience strategies among healthcare workers following the earthquakes.

**Methods:**

Following PRISMA 2020 and Joanna Briggs Institute guidance, we conducted a mixed-methods systematic review with convergent segregated synthesis. Nine sources (PubMed, Scopus, Web of Science, PsycINFO, CINAHL, Cochrane Library, DergiPark, TR Dizin and Google Scholar) were searched for studies published between February 2023 and September 2025, supplemented by forward and backward citation chasing. Twenty studies met inclusion criteria (fifteen quantitative, five qualitative). Random-effects meta-analysis estimated pooled probable-PTSD prevalence and pooled resilience–distress correlations, with leave-one-out, risk-of-bias, instrument and population sensitivity analyses. Qualitative findings were synthesised thematically and integrated with quantitative results.

**Results:**

Pooled prevalence of probable PTSD was 36.3% (95% CI 28.2–45.4), with high heterogeneity (I^2^ = 86.2%). Prevalence was 43.8% (95% CI 36.1–51.9) among direct survivors and 19.5% (95% CI 3.5–61.8) among deployed responders, but the latter was imprecise and is not interpreted as an exposure effect. Measurement was one apparent contributor: two studies applying PCL-5 ≥ 47 agreed closely (39.0%, I^2^ = 0.0%), but two applying IES-R ≥ 33 diverged widely (8.1 and 47.7%); measurement could not be separated from exposure, timing or sampling. Higher resilience, hardiness and adaptive coping were consistently associated with lower traumatic stress (pooled r = −0.42, 95% CI − 0.52 to −0.31, k = 4). Structural models indicated direct and coping-mediated associations, while fatigue and unmet basic needs predicted worse outcomes. Qualitative synthesis identified acute distress, secondary traumatisation, spirituality-based meaning-making, peer support and psychological distancing.

**Conclusion:**

Healthcare workers following the Kahramanmaraş earthquakes experienced high levels of trauma-related distress, particularly those with direct exposure. Resilience and adaptive coping were consistently inversely associated with distress, although cross-sectional designs cannot establish direction of effect, and organizational conditions were strongly implicated. Disaster preparedness and recovery efforts must integrate individual, social, and structural interventions to safeguard healthcare workers’ mental health.

**Systematic review registration:**

https://www.crd.york.ac.uk/PROSPERO/view/CRD420251247026, identifier CRD420251247026.

## Introduction

Natural disasters can precipitate profound and long-lasting psychological consequences in affected populations (Esterwood and Saeed, 2020). Earthquakes in particular are often followed by elevated rates of post-traumatic stress disorder (PTSD), depression, and anxiety among survivors (Çınaroğlu et al., 2025). Frontline health professionals constitute a uniquely vulnerable group in such disasters (Fredricks et al., 2017). Not only may they experience the disaster’s devastation first-hand as victims, but they are simultaneously tasked with responding to the emergency and caring for others—a dual exposure to trauma that heightens their psychological risk (Sehlikoğlu et al., 2023). Research has shown that healthcare workers involved in disaster response face intense stressors: they must make critical decisions and treat the injured amid chaos, all while potentially dealing with personal losses and danger to themselves (Alavi et al., 2023). In past earthquakes, health workers have reported high levels of acute stress (Wee and Myers, 2013), fear (Sultan et al., 2020), and grief (Rabow et al., 2021); many neglect their own emotional needs in the effort to help patients, which can lead to significant mental health problems over time (Naushad et al., 2019). This dual burden of personal trauma and caregiver stress is sometimes termed secondary traumatization, reflecting the indirect trauma of witnessing others’ suffering in addition to one’s own (Greinacher et al., 2019). In short, when disasters strike, those on the front lines of the health system may face compounded psychological threats—both direct and vicarious—that warrant dedicated attention.

The 2023 Kahramanmaraş earthquakes underscore these concerns. Striking southeastern Türkiye on February 6, 2023, with magnitudes 7.7 and 7.6, the twin earthquakes rank among the most devastating natural disasters in the country’s history (Karray et al., 2024). They caused widespread destruction across 11 provinces, killing approximately 50,000 people and injuring over 200,000 (Orak et al., 2023). Tragically, this toll included hundreds of healthcare workers—an estimated 505 health personnel lost their lives—and numerous hospitals and clinics were damaged or destroyed (Tayfur et al., 2024). The disaster thus not only created an immense need for medical response, but also directly struck the healthcare workforce and infrastructure. Health professionals in the region often found themselves working around the clock in makeshift conditions, all while coping with personal trauma, bereavement, and unsafe living conditions (Cecen Celik and O'Reilly, 2025). Contemporary accounts described physicians and nurses treating mass casualties despite their own homes being reduced to rubble and their families unaccounted for (Özbek Güven et al., 2024). Such conditions placed extraordinary psychological strain on health workers, raising urgent questions about their mental well-being in the aftermath.

Understanding the mental health impact on frontline health workers following the Kahramanmaraş earthquakes is critical for several reasons. First, from a humanitarian and ethical standpoint, these individuals deserve the same care and attention that they provide to others; their suffering should not be overlooked due to assumptions of “professional resilience.” Second, impaired mental health among healthcare providers can undermine the health system’s recovery—burnout, PTSD, or depression may affect job performance, workforce retention, and the quality of patient care over the long term. Finally, studying this group provides insights into how dual exposure to personal and occupational trauma operates, informing preparedness for future disasters. Early studies after the 2023 earthquakes hinted at a substantial psychological toll: for example, one survey of hospital staff in an affected city found that over one-third met criteria for probable PTSD just two months post-disaster (Aksoy et al., 2025). Risk appeared highest in those who had suffered personal losses (e.g., home damage or bereavement) in addition to working on the response. Such findings reinforce the need for a comprehensive synthesis of what has been learned about these outcomes and the factors that shape them.

This article therefore presents a mixed-methods systematic review and meta-analysis of the psychological impact and resilience strategies among health workers following the 2023 Kahramanmaraş earthquakes. A mixed-methods approach is warranted given the complex, multifaceted nature of this issue. Quantitative data (e.g., prevalence of PTSD or correlations of resilience with mental health) can delineate the scope and magnitude of the problem, while qualitative evidence (e.g., personal narratives of coping) provides depth and context, capturing nuances that statistics alone might miss. Integrating both strands allows us to bridge numbers with narratives, yielding a richer understanding that can better inform practice. This approach aligns with emerging trends in resilience research, where scholars recognize that purely quantitative syntheses may overlook experiential factors, and purely qualitative reviews may lack generalizability. By combining the two, we aim to overcome these limitations and produce a more holistic picture of health workers’ psychological experiences post-disaster.

In our review, we specifically examine both individual-level and system-level influences on health workers’ mental health. At the individual level, we focus on personal resilience factors (such as psychological hardiness, coping skills, and social support networks) that might buffer against trauma. These factors have been widely theorized as protective; indeed, previous evidence suggests that strong resilience and adaptive coping are associated with lower PTSD risk following trauma. We anticipate, for example, that health workers with higher resilience or effective coping strategies will report fewer post-traumatic symptoms, consistent with the broader literature. At the system or organizational level, we consider workplace and contextual factors—for instance, workload, shift length, resource availability, and institutional support. Disaster settings often impose extreme working conditions: long hours, insufficient rest, high patient loads, supply shortages, and chaotic environments (Harrell et al., 2020). Such stressors can compound trauma exposure and have been identified as risk factors for PTSD and burnout in healthcare providers even outside of disaster contexts (Brooks et al., 2016). We hypothesize that health workers operating under severe resource constraints or lacking organizational support will experience worse psychological outcomes. By investigating both levels of influence, our review acknowledges that resilience is not just an intrinsic personal trait, but also a product of one’s environment. A supportive work context may bolster individual resilience, whereas an adverse context can overwhelm even the most resilient individuals.

On the basis of this reasoning we advanced three hypotheses. First, we hypothesised that the prevalence of probable PTSD among health professionals exposed to the Kahramanmaraş earthquakes would substantially exceed the rates reported for medical responders to earthquakes internationally. Second, we hypothesised that resilience, hardiness and adaptive coping would show a consistent inverse association with traumatic stress across samples and operationalisations, and that this association would be at least moderate in magnitude. Third, we hypothesised that organisational and contextual conditions—workload, fatigue, resource availability and institutional support—would be associated with psychological outcomes independently of individual resilience, such that adverse working conditions would predict worse outcomes even among those with greater personal resources.

## Methods

### Study design

This review was conducted as a mixed-methods systematic review and meta-analysis examining the psychological impact and resilience strategies of health workers following the 2023 Kahramanmaraş earthquakes. It was developed in accordance with the Preferred Reporting Items for Systematic Reviews and Meta-Analyses (PRISMA) 2020 guidelines and followed the Joanna Briggs Institute (JBI) methodology for mixed-methods reviews. A convergent segregated approach was used, whereby quantitative and qualitative evidence were synthesized independently and later integrated during interpretation. Both quantitative (e.g., cross-sectional surveys) and qualitative (e.g., interview-based) studies were eligible for inclusion. The review protocol was prospectively registered in the International Prospective Register of Systematic Reviews (PROSPERO; registration no. CRD420251247026) prior to study selection. The completed PRISMA checklist is provided in the Supplementary Material 2.

### Search strategy

A comprehensive literature search was performed to identify studies of health professionals’ psychological outcomes and resilience following the 2023 Kahramanmaraş earthquakes. Nine sources were searched: PubMed, Scopus, Web of Science Core Collection, PsycINFO, CINAHL, the Cochrane Library, DergiPark, ULAKBİM TR Dizin, and Google Scholar (first 200 records by relevance). The search covered the period from 6 February 2023 to 30 September 2025, and was updated in September 2026 prior to resubmission. Only studies published in English or Turkish were eligible; publications in other languages were excluded, and no filter was applied on indexing status, publication venue or sample size.

The search combined four concept blocks: the disaster (Kahramanmaraş, Pazarcık, Elbistan, Türkiye earthquake, Turkey earthquake, earthquake*); the population; psychological outcomes (mental health, PTSD, post-traumatic stress, depression, anxiety, psychological distress, secondary traumatic stress, burnout, compassion fatigue, sleep); and resilience and coping (resilience, hardiness, coping, post-traumatic growth). The population block was deliberately specified more broadly than the eligibility criteria, so that population-based exclusions would be documented rather than implicit. In addition to health-worker terms (healthcare worker*, health personnel, medical staff, nurse*, physician*, doctor*, midwife*, allied health), it included first-responder and emergency-personnel terms in both languages: first responder/ilk müdahale ekipleri, firefighter/itfaiyeci, search and rescue/arama kurtarma, AFAD personnel, disaster responder/afet müdahale, emergency medical services/acil sağlık hizmetleri, paramedic/paramedik, emergency personnel/acil personel, and rescue worker/kurtarma görevlisi. Records retrieved through these terms were screened against the population criterion and, where ineligible, recorded in Supplementary Table S1 rather than discarded silently.

Three supplementary procedures were used. Reference lists of all included studies were screened, forward citation searching was performed in Web of Science and Google Scholar for every included study, and the reference list supplied by a peer reviewer during the review of this manuscript was screened as an additional source. These streams are shown separately in Figure 1. All records were imported into reference management software and deduplicated before screening. Full database-specific strategies are provided in Supplementary Table S2.

**Figure 1:**
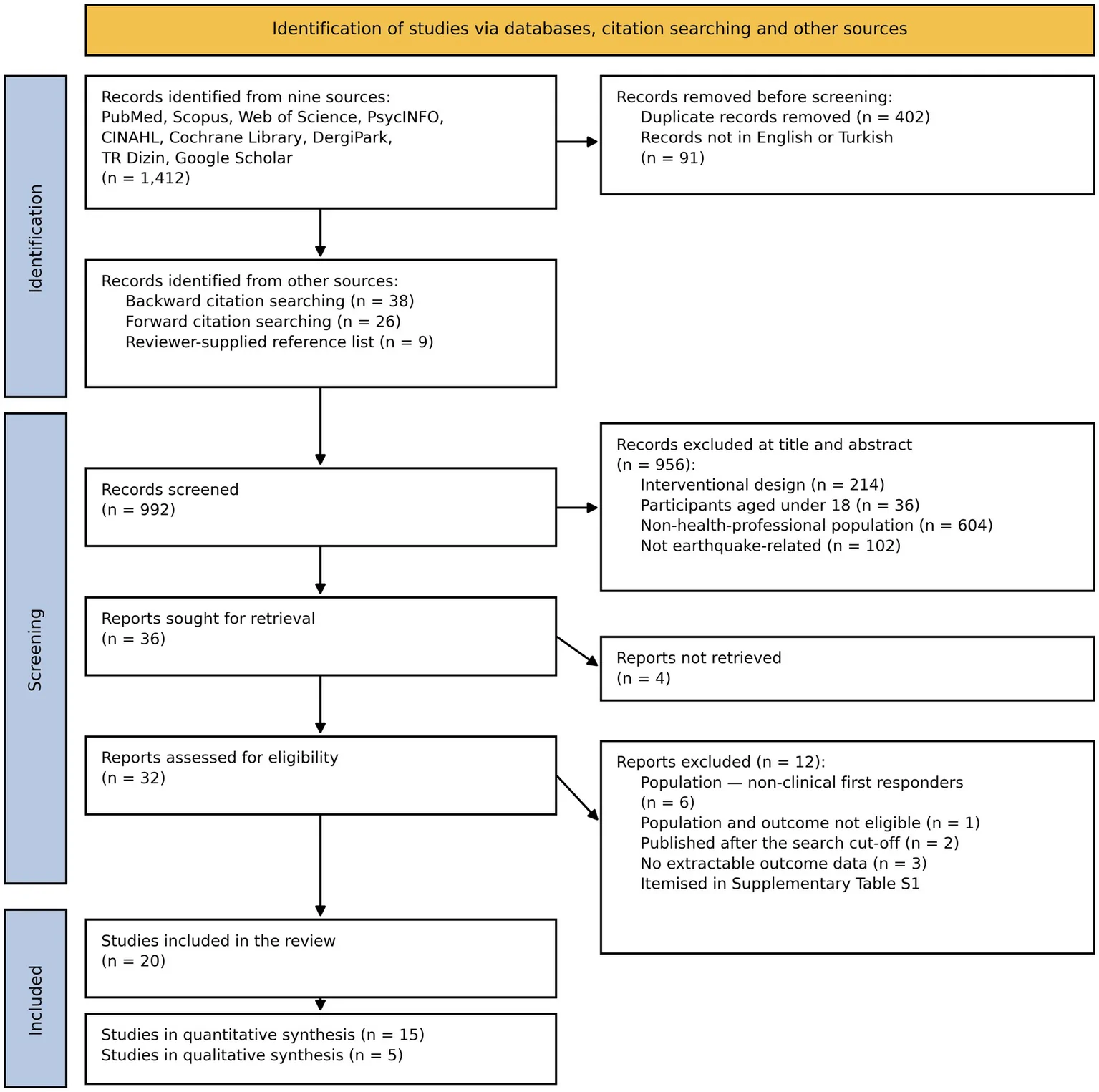
PRISMA 2020 flow diagram of study identification, screening and inclusion.

### Study selection

Titles and abstracts of retrieved records were screened independently by two reviewers against the inclusion criteria. Eligible participants were health professionals—physicians, dentists, nurses, midwives, paramedics and other emergency medical services clinicians, allied health professionals, mental health professionals, and hospital-based health support personnel—who were (i) resident or employed in one of the eleven provinces affected by the 6 February 2023 Kahramanmaraş earthquakes at the time of the disaster, (ii) deployed into the affected region as part of the organised health response, or (iii) providing clinical care to earthquake survivors outside the affected region. Studies were further required to (iv) report at least one psychological outcome (post-traumatic stress, depression, anxiety, secondary traumatic stress, burnout, sleep disturbance, or general psychological distress) or a resilience or coping construct, assessed with a named instrument or through a defined qualitative method, and (v) employ a quantitative, qualitative or mixed-methods design.

Studies of non-clinical first responders—firefighters, search-and-rescue teams, AFAD personnel, police, military personnel, and untrained volunteers—were not eligible unless outcomes for a health-professional subgroup were reported separately. This boundary was set for three reasons. First, the constructs organising this review—secondary traumatisation arising from sustained therapeutic contact with survivors, compassion satisfaction and compassion fatigue, and moral injury arising from an inability to deliver adequate clinical care—are properties of a continuing caregiving relationship rather than of time-limited operational deployment. Second, the analytic core of the review is the contrast between health professionals who were themselves survivors and those who were deployed; because non-clinical responders are almost entirely deployed personnel with different selection, training and pre-existing occupational trauma profiles, including them would confound occupation with exposure. Third, heterogeneity across the eligible literature is already extreme, and further broadening of the population would increase dispersion without a corresponding gain in interpretability. The consequences of this boundary were tested empirically in a prespecified sensitivity analysis, reported below. Studies excluded on population grounds are listed individually, with citations, in Supplementary Table S1.

Full-text articles of all potentially eligible studies were assessed, in the original language where necessary, by both reviewers. No minimum sample size was applied and no study was excluded on the basis of methodological quality; quality was instead incorporated through risk-of-bias sensitivity analysis. Disagreements were resolved through discussion or consultation with a third reviewer. The study selection process is summarised in a PRISMA 2020 flow diagram (Figure 1), and studies assessed at full text but excluded are itemised with reasons in Supplementary Table S1.

### Data extraction

Key data were extracted from each included study using a standardized form tailored to study type. For quantitative studies, we extracted information on study design and setting, participant characteristics (e.g., profession, sample size), measures of psychological outcomes (such as prevalence or mean scores of PTSD, depression, anxiety), and any reported associations between resilience factors and mental health outcomes (e.g., correlation coefficients or effect sizes). For qualitative studies, we captured details on the study context and population, data collection methods (e.g., interview or focus group guide), and the main findings related to psychological impact and resilience (including identified themes and illustrative quotations). Data extraction was performed by one reviewer and verified by a second to ensure accuracy.

Record management, deduplication and the preparation of the extraction forms were supported by a research assistant, who also carried out first-pass data extraction for the studies added at revision. All extracted values were subsequently checked against the source articles independently by both authors, and any discrepancy between the first-pass extraction and the source was resolved by returning to the published report. Where the text and tables of a source article disagreed, the tables were treated as authoritative and the disputed figure was not extracted.

### Quality and bias assessment

The methodological quality and risk of bias of the included studies were assessed using the Joanna Briggs Institute (JBI) critical appraisal tools appropriate to each study design. Quantitative studies, which were predominantly cross-sectional, were evaluated using the JBI Checklist for Analytical Cross-Sectional Studies, while qualitative studies were appraised using the JBI Checklist for Qualitative Research. Two reviewers independently assessed each study across domains including clarity of inclusion criteria, validity and reliability of exposure and outcome measurements, consideration of potential confounding, and appropriateness of statistical or analytic methods. Discrepancies between reviewers were resolved through discussion and consensus.

All twenty included studies were appraised. The JBI Analytical Cross-Sectional Studies checklist was applied to the fourteen cross-sectional quantitative studies, the JBI Case–Control checklist to the one case–control study (Emirza et al., 2025), and the JBI Qualitative Research checklist to the five qualitative studies. Studies added during revision were appraised using the same instruments and the same transparent overall-rating rule as those included in the original synthesis. Full item-level appraisals are provided in Supplementary Tables S3a–c.

No study was excluded on the basis of quality assessment alone. Instead, risk-of-bias judgments were taken into account when interpreting the findings, particularly in relation to common design limitations such as non-probability sampling and incomplete control of confounding. Detailed item-level risk-of-bias assessments for all included studies are provided in the Supplementary Tables S3a–c. Sensitivity analyses were conducted to examine the robustness of the quantitative findings to the inclusion of studies with higher risk of bias.

### Statistical analyses

#### Qualitative data synthesis

Qualitative findings were synthesized using a thematic synthesis approach. This involved coding the textual data from each qualitative study line-by-line and grouping similar codes into higher-order themes that characterize health workers’ psychological experiences and resilience strategies. The thematic synthesis preserved original participant perspectives while allowing identification of common patterns across studies. Where available, multiple reviewers discussed and refined the coding and theme development to enhance reliability of the synthesis. Qualitative and quantitative results remained separate during analysis and were brought together in the interpretation phase, consistent with the convergent segregated design.

#### Quantitative data synthesis

Quantitative outcomes were analyzed using meta-analytical techniques. For prevalence outcomes (e.g., prevalence of PTSD, depression, or anxiety symptoms among health workers), we performed a random-effects meta-analysis to calculate pooled prevalence estimates and 95% confidence intervals. A random-effects model was chosen *a priori* given the expectation of between-study heterogeneity in populations and measures. For bivariate associations (e.g., correlations between resilience scores and psychological distress levels), Pearson’s correlation coefficients (r) were extracted from each study and transformed to Fisher’s z scores for meta-analysis. We then pooled these z-transformed correlations using a DerSimonian-Laird random-effects model and back-transformed the overall effect size to an r value for interpretability. In cases where included quantitative studies reported complex analyses such as structural equation models (SEM) to examine relationships among variables, those results could not be synthesized quantitatively; instead, the SEM findings were narratively summarized in the review.

#### Heterogeneity and additional analyses

Statistical heterogeneity across studies was assessed with the I^2^ statistic, with values above approximately 50% considered indicative of substantial heterogeneity. We also examined Cochran’s Q test for heterogeneity (with a significance level of *p* < 0.10). When moderate or high heterogeneity was detected, potential sources were explored via subgroup analyses (for example, analyzing subsets of studies by type of outcome, location, or timing of assessment) and by examining study-level differences. To evaluate the robustness of the meta-analytic results, we conducted sensitivity analyses. These included re-running meta-analyses after excluding studies at high risk of bias and checking whether any single study unduly influenced the pooled estimates (by omitting each study in turn). All quantitative meta-analyses were conducted in R version 4.3.1 using the “meta” and “metafor” packages. Prevalence estimates were pooled using logit-transformed proportions, and correlations were synthesized using Fisher’s z-transformed Pearson coefficients. Thematic synthesis of qualitative data was conducted manually using structured coding in Microsoft Excel. Structural equation model findings from primary studies were narratively summarized based on reported path coefficients and model fit indices. The findings from quantitative and qualitative syntheses were finally compared and integrated in the overall interpretation, allowing us to draw comprehensive conclusions about the psychological impact on health workers and the resilience strategies they employed after the disaster.

Four sensitivity analyses were specified for the revision. First, leave-one-out analysis was performed for both the prevalence and the correlation syntheses to identify disproportionately influential studies. Second, each synthesis was re-run after excluding studies rated at moderate-to-high risk of bias on the JBI appraisal. Third, a first-responder sensitivity analysis was conducted in which studies of non-clinical disaster responders—excluded from the primary synthesis on population grounds—were added to both models, in order to establish empirically whether the population boundary materially affected the pooled estimates. Fourth, because the instruments and thresholds used to define probable PTSD varied across the included studies (IES-R ≥ 33, TSSS-5 ≥ 25, and PCL-5 ≥ 47), a subgroup analysis by instrument was conducted to assess ascertainment effects. Results of all sensitivity analyses are reported in Supplementary Table S4.

Because several studies reported more than one eligible coefficient, a single effect per independent sample was entered into the correlation synthesis in order to preserve independence, following a rule fixed before the analysis was run. Where a study reported the same resilience measure against two traumatic stress outcomes, the coefficients were averaged; this applied to Koca et al. (2025), whose hardiness measure was reported against the TSSS (r = −0.281) and the PETSSS (r = −0.294) and was entered as r = −0.288. Where a study reported both a resource and a risk construct, only the resource coefficient was entered, since entering both would duplicate the sample; this applied to Onat et al. (2025), for whom the effective-coping coefficient was entered and the ineffective-coping coefficient was not. Studies whose outcome was a positive occupational construct rather than a traumatic stress outcome were not entered, which excluded the hardiness-with-compassion-satisfaction coefficient reported by Kınık et al. (2024). Four independent samples therefore contributed to the pooled correlation. All seven eligible coefficients are displayed in Table 1, with those entering the pooled model identified.

**Table 1:** Associations between resilience-related measures and traumatic stress outcomes, showing which coefficients were entered into the pooled analysis.

Author (Year)	Population	Resilience/Resource measure	Traumatic stress outcome	r	Direction	Entered in pooled analysis
Polat H. et al. (2025)	Survivor HCWs	Brief Psychological Resilience Scale (BPRS)	TSSS-5 total	−0.523	Inverse	Yes
Koca et al. (2025)	Survivor HCWs	Personal Hardiness Scale (PHS)	TSSS total	−0.281	Inverse	Averaged with the row below and entered as −0.288
Koca et al. (2025)	Survivor HCWs	Personal Hardiness Scale (PHS)	PETSSS total	−0.294	Inverse	Averaged with the row above and entered as −0.288
Çobanoğlu and Oğuzhan (2024)	Nurses (affected hospitals)	Brief Resilience Scale (BRS)	Rumination (RTSQ)	−0.477	Inverse	Yes
Onat et al. (2025)	Vicarious exposure HCWs	Effective coping styles	STSS total	−0.380	Inverse	Yes
Onat et al. (2025)	Vicarious exposure HCWs	Ineffective coping styles	STSS total	+0.480	Positive	No—risk rather than resource construct, and same sample as the row above
Kınık et al. (2024)	Deployed responders	Hardiness	Compassion satisfaction (ProQOL)	+0.571	Positive	No—outcome is not a traumatic stress measure

## Results

### Study selection and characteristics

The updated search and screening process yielded twenty studies that met the inclusion criteria. Of these, fifteen employed quantitative designs (fourteen cross-sectional, including two that incorporated structural equation modelling, and one case–control study) and five used qualitative approaches. Eight studies were added during revision following an expanded search: six quantitative reports (Çifçi and Kılınç, 2024; Emirza et al., 2025; Gökçek et al., 2024; Satılmış et al., 2024; Şehlikoğlu et al., 2024; Topkara et al., 2024) and two qualitative reports (Deniz Doğan et al., 2024; Mert and Köksal, 2025). Studies assessed at full text and excluded are itemised with reasons in Supplementary Table S3; the largest single category of exclusion was population, comprising studies of firefighters, search-and-rescue teams and mixed disaster-responder samples in which no health-professional subgroup was reported separately.

All included studies were conducted in Türkiye and published between 2023 and 2025. Sample sizes ranged from 15 to 642 participants. The included studies span three exposure contexts: health professionals who directly experienced the earthquakes in the affected provinces; health professionals deployed into the disaster region as part of the organised response; and health professionals with indirect or vicarious exposure through caring for survivors outside the affected zone. In the one case–control study, only the deployed case group met the population criteria; the non-deployed control group is reported as a comparator and contributes to no pooled estimate. Post-traumatic stress was assessed with the Impact of Event Scale–Revised (IES-R), the Traumatic Stress Symptom Scale-5 (TSSS-5), the PTSD Checklist for DSM-5 (PCL-5), a brief PTSD short scale, or the Secondary Traumatic Stress Scale (STSS). Resilience and related constructs were operationalised using the Brief Resilience Scale, the Brief Psychological Resilience Scale, hardiness scales, disaster-specific resilience questionnaires, and coping inventories. The five qualitative studies used in-depth interviews or focus groups with phenomenological, thematic or content analysis.

This variability in instrument, threshold, exposure context and timing was substantial and, as reported below, proved consequential for the pooled estimates. We therefore conducted separate quantitative syntheses for prevalence and correlation outcomes alongside the qualitative thematic synthesis, and integrated the strands at the interpretation stage in accordance with the convergent segregated design. Characteristics of all included studies are summarised in Table 2.

**Table 2:** Characteristics of studies included in the systematic review (*N* = 20).

Author (year)	Country	Design	Sample (*n*)	Population/exposure type	Time post-EQ	Measures	Key outcomes
Aksoy et al. (2025)	Türkiye	Cross-sectional	522	Survivor HCWs (affected region)	~5–8 mo	IES-R, ESCSS	PTSD prevalence 47.7% (IES-R ≥ 33); coping patterns
Polat H. et al. (2025)	Türkiye	Cross-sectional	313	Survivor HCWs	~6–9 mo	TSSS-5, BPRS	PTSD prevalence 39.6%; resilience r = −0.52 with TSSS
Polat I. et al. (2025)	Türkiye	Cross-sectional	62	Deployed HCPs	~1–2 mo	IES-R, PSS	Probable PTSD 8.1% (IES-R ≥ 33); workload predictors
Koca et al. (2025)	Türkiye	Cross-sectional + SEM	315	Survivor HCWs	~6 mo	PETSSS, TSSS, PHS, CRI	Resilience → coping → ↓ traumatic stress (mediation)
Kiymis and Yuce (2025)	Türkiye	Cross-sectional + SEM	642	Survivor HCWs	~4–8 mo	DRT, PTSD-12, PHQ-9, SDQ	Resilience ↓ PTSD/depression; ↑ sleep disturbance
Onat et al. (2025)	Türkiye	Cross-sectional	243	Indirect exposure HCWs (secondary traumatic stress)	NR	STSS, BAI, BDI, Coping Scale	STSS correlated with anxiety/depression and coping
Çobanoğlu and Oğuzhan (2024)	Türkiye	Cross-sectional	225	Nurses (affected hospitals)	~6 mo	BRS, RTSQ	Resilience r = −0.48 with rumination
Kınık et al. (2024)	Türkiye	Cross-sectional	400	Deployed responders (Red Crescent)	~3–6 mo	ProQOL, Hardiness	Hardiness r = 0.57 with compassion satisfaction
Dağlar et al. (2025)	Türkiye	Cross-sectional	64	Deployed neurosurgeons	~1–3 mo	CESQ	Religious coping ↔ positive reappraisal (r = 0.40)
Emirza et al. (2024)	Türkiye	Qualitative (phenomenology)	18	Deployed volunteer nurses	~2–4 mo	Semi-structured interviews	Coping themes: spirituality, support, avoidance
Koksal et al. (2025)	Türkiye	Qualitative	15	Survivor nurses	~14 mo	In-depth interviews	Trauma, coping, work-performance impacts
Uysal et al. (2025)	Türkiye	Qualitative	20	Deployed emergency nurses	Immediate–early	Focus groups	System barriers, peer support, personal strategies
Satılmış et al. (2024)	Türkiye	Cross-sectional, multicentre	79	HCWs assigned to the earthquake zone (49.4% physicians, 50.6% nurses)	~2 mo after assignment	PCL-5	Probable PTSD 37.9% (30/79, PCL-5 ≥ 47); female sex, nursing role and prior level-1 hospital work independent risk factors
Şehlikoğlu et al. (2024)	Türkiye	Cross-sectional	175	Mixed: 149 employed in the zone pre-EQ, 26 assigned post-EQ	~6 mo	PCL-5, PHQ-9, GAD-7, MBI, PTGI, SF-12	Probable PTSD 39.4% (69/175); depression 30.3%; anxiety 31.4%; moderate-to-high PTG 37.7%; high emotional exhaustion 13.7%
Çifçi and Kılınç (2024)	Türkiye	Cross-sectional	299	Survivor HCWs, Adıyaman (94% experienced the EQ; 85.3% home damaged)	~10–11 mo	PTSD Short Scale, BAI, BDI, PSQI	Medians: PTSD 18.0 (cut-off 24), BAI 19.0, BDI 16.0, PSQI 9.0 (poor sleep); PTSD–PSQI r = 0.579
Topkara et al. (2024)	Türkiye	Cross-sectional	475	HCWs in Hatay (65.1% midwives, 18.1% nurses)	~3–6 mo	STSS, Compassion Fatigue Scale-SF	STSS 55.6 ± 12.3; CF 71.7 ± 28.3; STSS–CF r = 0.739; higher STS with physical injury, home destruction, bereavement
Emirza et al. (2025)	Türkiye	Case–control	146 + 143	HCWs deployed to the zone and returned vs. never deployed; neither group directly exposed	~14–16 mo	CF-SS, CESS	Deployed group higher compassion fatigue (44.40 ± 24.82 vs. 40.72 ± 26.79) and occupational burnout (27.45 ± 16.13 vs. 25.42 ± 17.45); coping profiles broadly similar
Gökçek et al. (2024)	Türkiye	Cross-sectional	159	HCWs treating survivors transferred out of the zone (indirect exposure)	~7 days onward	BAI, BDI	Anxiety (BAI > 7) 46.5%; depression (BDI ≥ 17) 22.6%; both higher in women
Mert and Köksal (2025)	Türkiye	Qualitative (descriptive)	15	Volunteer nurses deployed to the zone	5–15 days post-mission	Semi-structured interviews	Moral obligation, preparation and coordination failures, workload management, post-mission anxiety and burnout
Deniz Doğan et al. (2024)	Türkiye	Qualitative (phenomenology)	30	Survivor-region nurses caring for survivors (Adana, Şanlıurfa)	~5–8 wk	Semi-structured interviews	Selflessness, obligation, tiredness, satisfaction; managerial and personal difficulties in care delivery

### Prevalence of PTSD and traumatic stress symptoms

Ten of the quantitative studies reported outcomes related to post-traumatic stress or traumatic stress among health professionals in the aftermath of the earthquakes (Table 3). Five of these provided explicit prevalence estimates of probable PTSD based on predefined score cut-offs, while the remainder reported traumatic stress as continuous symptom severity or as median scale scores. Table 3 presents the studies contributing dichotomous data together with those reporting continuous outcomes, and shows the variation in instrument and threshold across the evidence base.

**Table 3:** Prevalence of probable PTSD and traumatic stress among health professionals after the 2023 Kahramanmaraş earthquakes.

Author (year)	Population/exposure type	Measure	Cutoff	n/N (%)	Time post-EQ
Aksoy et al. (2025)	Survivor HCWs (affected region)	IES-R	≥33	249/522 (47.7%)	~5–8 mo
Polat H. et al. (2025)	Survivor HCWs	TSSS-5	≥25	124/313 (39.6%)	~6–9 mo
Polat I. et al. (2025)	Deployed HCPs	IES-R	≥33	5/62 (8.1%)	~1–2 mo
Polat I. et al. (2025)	Deployed HCPs	IES-R	>24	18/62 (29.0%)	~1–2 mo
Satılmış et al. (2024)	HCWs assigned to the zone	PCL-5	≥47	30/79 (37.9%)	~2 mo
Şehlikoğlu et al. (2024)	Mixed (85% pre-EQ resident)	PCL-5	≥47	69/175 (39.4%)	~6 mo
Çifçi and Kılınç (2024)	Survivor HCWs (Adıyaman)	PTSD Short Scale	24	Median 18.0 (IQR 11.0–25.0); proportion above cut-off not reported	~10–11 mo
Onat et al. (2025)	Indirect exposure HCWs	STSS	—	Mean 38.9 ± 13.2	NR
Koca et al. (2025)	Survivor HCWs	TSSS/PETSSS	—	Continuous scores	~6 mo
Topkara et al. (2024)	HCWs in Hatay	STSS	—	Mean 55.6 ± 12.3	~3–6 mo
Gökçek et al. (2024)	Indirect exposure HCWs	BAI/BDI	>7/≥17	Anxiety 74/159 (46.5%); depression 36/159 (22.6%)	~7 days onward

Probable-PTSD prevalence is not directly comparable across the IES-R, TSSS-5 and PCL-5 cut-offs applied here.

Among health workers who were direct survivors of the earthquakes, the prevalence of probable PTSD was notably high several months after the disaster. For example, Aksoy et al. (2025) reported that 47.7% of participants met the IES-R cutoff (score ≥33) for probable PTSD at approximately 5–8 months post-earthquake. Similarly, Polat H. et al. (2025) found a 39.6% prevalence of probable PTSD (using TSSS-5 ≥ 25 as the threshold) at about 6–9 months post-disaster. These findings indicate a substantial and persisting psychological burden among directly exposed health workers in the half-year following the earthquakes.

In contrast, markedly lower PTSD prevalence was observed among deployed responders who assisted in the disaster region but did not themselves survive the earthquakes. Polat I. et al. (2025) found that 8.1% of such deployed health professionals met the criteria for probable PTSD (IES-R ≥ 33) within one to two months of their deployment. However, when using a more lenient threshold for symptoms (IES-R > 24, indicating any clinically elevated traumatic stress), nearly one-third of the responders (29.0%) exhibited high levels of distress. This finding suggests that even without direct personal exposure to the earthquake, a considerable minority of disaster responders experienced significant psychological symptoms in the immediate aftermath.

Studies of indirect exposure (secondary trauma) further showed that vicarious exposure was associated with substantial stress, although formal PTSD cut-off scores were not always applied. Onat et al. (2025) documented elevated Secondary Traumatic Stress Scale scores in health workers who were caring for earthquake survivors outside the disaster zone (e.g., an average STSS score around 39 on a scale where this level is clinically noteworthy). Likewise, Koca et al. (2025) reported high traumatic stress symptom levels among health workers who survived the earthquake, using continuous post-traumatic stress scales rather than a dichotomous PTSD diagnosis. In their sample, a large proportion of participants scored in a range considered to be of clinical concern, even if not categorized as “PTSD positive.” Taken together, these results suggest that both direct and indirect exposure to the disaster were associated with significant psychological stress reactions in health workers, although the apparent prevalence and severity varied by exposure type, timing of assessment, and measurement approach.

Five studies reported dichotomous probable-PTSD data and were meta-analysed: Aksoy et al. (2025), Polat H. et al. (2025; 124/313, TSSS-5 ≥ 25), Polat I. et al. (2025; 5/62, IES-R ≥ 33), Satılmış et al. (2024), and Şehlikoğlu et al. (2024). Çifçi and Kılınç (2024) reported median scale scores rather than proportions above cut-off and could not contribute. Random-effects pooling of logit-transformed proportions yielded an overall probable-PTSD prevalence of 36.3% (95% CI 28.2–45.4), with high between-study heterogeneity (I^2^ = 86.2%, Q = 29.04, df = 4, *p* < 0.001). Roughly one in three disaster-exposed health professionals therefore screened positive for clinically significant trauma-related symptoms, although the dispersion is such that this should be read as an order of magnitude rather than a precise figure.

Subgroup analysis by exposure context produced a less clear-cut picture than an analysis restricted to the originally included studies would have suggested. Among health professionals who were direct earthquake survivors, pooled prevalence was 43.8% (95% CI 36.1–51.9; I^2^ = 80.6%). Among deployed personnel it was 19.5% (95% CI 3.5–61.8; I^2^ = 92.8%), an interval so wide as to be uninformative. Şehlikoğlu et al. (2024) (39.4%) drew on a mixed sample, 85% of whom were employed in the affected provinces before the earthquakes, and is not assigned to either stratum. The two deployed studies differ markedly—8.1% in Polat I. et al. (2025) against 37.9% in Şehlikoğlu et al. (2024)— and differ simultaneously in instrument and threshold (IES-R ≥ 33 versus PCL-5 ≥ 47), in deployment intensity, and in the interval between deployment and assessment. The apparent gradient from deployed responders to direct survivors is therefore confounded with ascertainment and cannot be read as a straightforward effect of exposure type.

Subgroup analysis by instrument was conducted to examine whether ascertainment contributed to the dispersion, and gave a mixed answer. The two studies applying PCL-5 ≥ 47 pooled at 39.0% (95% CI 33.2–45.1) with I^2^ = 0.0%, agreeing closely despite differing in exposure stratum, region, sampling method and assessment interval. The two applying IES-R ≥ 33, however, reported 8.1 and 47.7% and pooled at 22.8% (95% CI 2.9–74.6) with I^2^ = 95.9%—a divergence as large as any in the dataset, between two studies using the same instrument at the same threshold. The single TSSS-5 study reported 39.6%. Measurement approach is therefore not sufficient to account for the observed dispersion, and these two subgroups point in opposite directions. Leave-one-out analysis identified Polat I. et al. (2025) as the single influential study: omitting it raised the overall estimate to 42.0% (95% CI 36.9–47.3) and reduced I^2^ from 86.2 to 61.6%. With five studies distributed across three instruments, three exposure categories and assessment intervals ranging from approximately one to eleven months, instrument, threshold, exposure, timing, sampling frame and setting are fully confounded with one another and cannot be separated. These subgroup estimates are therefore reported as descriptive, and no single study-level characteristic is identified as the dominant source of heterogeneity. Excluding studies rated at moderate-to-high risk of bias left the pooled estimate unchanged, since none of the five contributing studies received that rating. Figure 2 presents the individual study estimates and the pooled result.

**Figure 2:**
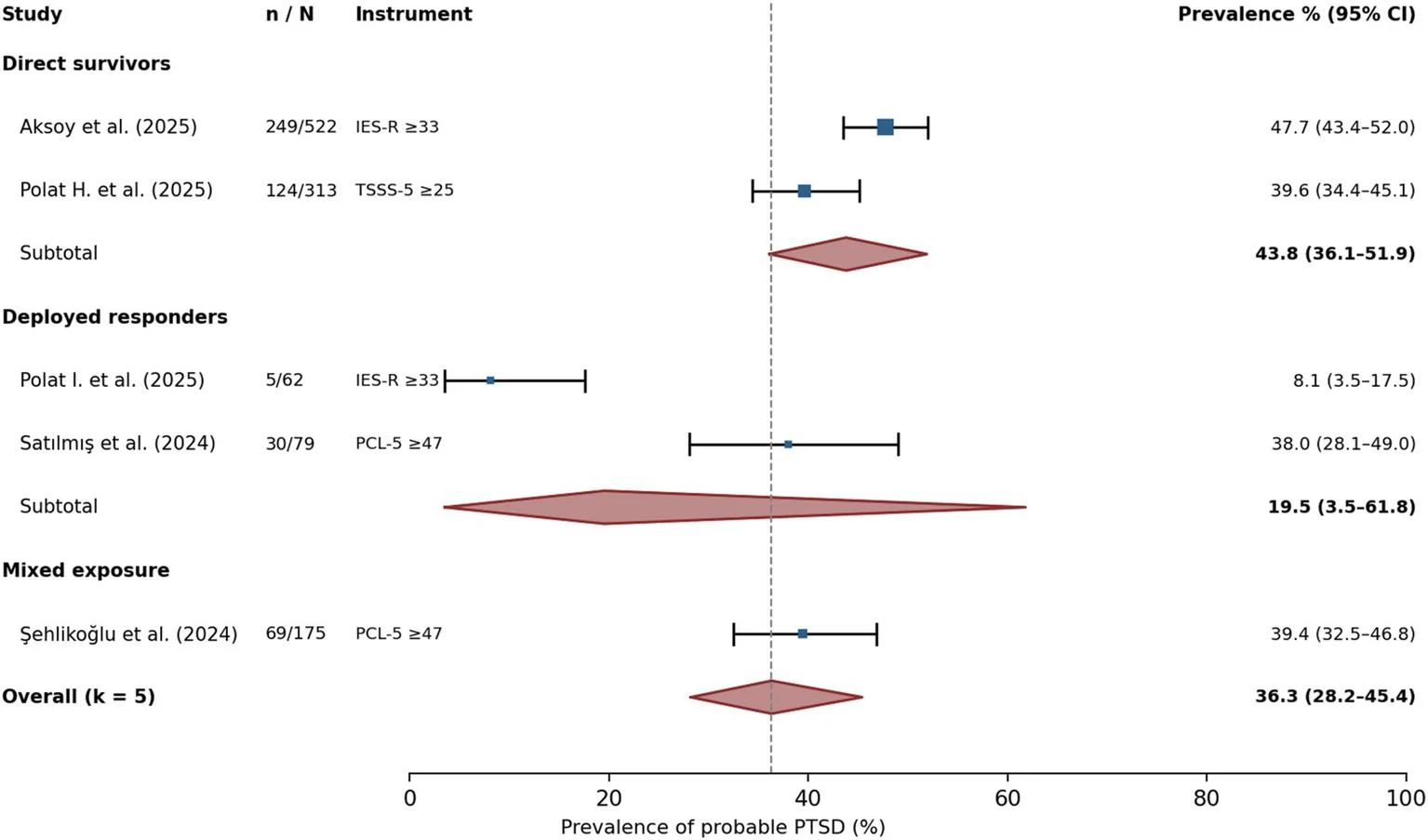
Forest plot of pooled probable PTSD prevalence among health professionals after the 2023 earthquakes, stratified by exposure type.

A first-responder sensitivity analysis was conducted by adding the two excluded non-clinical responder studies that report usable data (Bakirci et al., 2024, 36/223 above PCL-5 ≥ 41; İşeri and Baltacı, 2024, 60/170 on the PCL-5). The pooled prevalence fell to 31.5% (95% CI 23.2–41.3) and heterogeneity rose from I^2^ = 86.2 to 92.4% (k = 7); pooled alone, the two responder studies yielded 24.5% (95% CI 10.5–47.4; I^2^ = 94.6%). Broadening the population therefore lowered the estimate and worsened dispersion without improving interpretability, supporting the population boundary set *a priori*. Full sensitivity results are reported in Supplementary Table S4.

### Associations between resilience, hardiness and traumatic stress outcomes

Across the quantitative studies, higher levels of resilience-related resources were consistently associated with lower levels of traumatic stress and psychological distress in health workers (Table 1). Reported effect sizes for these relationships ranged from small-to-moderate to large, overall indicating a consistent inverse association between resilience-related measures and post-disaster mental health outcomes across different samples and operationalizations of resilience. Because all contributing studies were cross-sectional, these associations are reported without assumption as to their direction.

Focusing on direct earthquake survivors, Polat H. et al. (2025) documented a strong inverse relationship between psychological resilience and traumatic stress symptoms in health workers: resilience (measured by the Brief Psychological Resilience Scale) was negatively correlated with PTSD symptom severity (TSSS-5 scores) at r = −0.52. In other words, individuals reporting higher resilience also reported substantially fewer post-traumatic stress symptoms. Similarly, Koca et al. (2025) observed moderate negative correlations between personal hardiness and traumatic stress severity in a sample of survivor health workers. In that study, greater hardiness was significantly associated with lower trauma symptom scores on two different scales (correlations approximately r = −0.28 and r = −0.29 for two trauma measures), consistent with the notion that hardiness—a trait linked to resilience—accompanies less severe stress reactions, although a cross-sectional design cannot test buffering.

Inverse associations were also evident in specific subgroups and with related outcomes. In a study of nurses working at an affected hospital, Çobanoğlu and Oğuzhan (2024) found that higher resilience (measured by the BRS) was strongly correlated with lower rumination levels (r = −0.48). Rumination is a cognitive process associated in the wider literature with more severe and more prolonged PTSD symptoms, so this finding is consistent with resilience and rumination indexing partly overlapping processes, although the cross-sectional design does not permit that interpretation to be tested. Among health workers indirectly exposed to trauma through patient care, Onat et al. (2025) demonstrated that coping styles made a significant difference: effective (adaptive) coping was associated with lower secondary traumatic stress (r = −0.38), whereas ineffective (maladaptive) coping was associated with higher secondary stress (r = +0.48). In other words, those who used active and positive coping strategies had fewer secondary trauma symptoms, while those who coped through negative or avoidant strategies had elevated symptoms.

Resilience-related resources were also associated with positive outcomes. For example, Kınık et al. (2024) reported that psychological hardiness showed a strong positive correlation with compassion satisfaction (a measure of professional fulfillment) in disaster responders (r = +0.57). This indicates that higher hardiness scores co-occurred not only with lower distress but also with greater reported satisfaction and meaning in work carried out in high-stress disaster contexts.

Overall, the converging quantitative evidence indicates that resilience, hardiness and adaptive coping styles are consistently and inversely associated with traumatic stress among disaster-exposed health workers. There was some variability in the magnitude of correlations across studies, reflecting differences in specific measures, populations and contexts, but the direction of association was uniform. Notably, one particular coping mechanism, positive reappraisal (cognitively reframing the situation to find meaning or growth), was repeatedly associated with lower stress levels. Several studies that assessed coping reported that participants using positive reappraisal and related adaptive strategies scored lower on trauma-related measures. These are associations observed at a single time point, and the available designs cannot distinguish resilience buffering distress from distress eroding self-reported resilience.

To quantify the overall relationship between resilience-related resources and traumatic stress outcomes, we meta-analysed one coefficient from each independent sample reporting an eligible bivariate correlation, applying the selection rule described in the Methods. Four samples contributed: Polat H. et al. (2025, r = −0.523), Koca et al. (2025), Çobanoğlu and Oğuzhan (2024), and Onat et al. (2025). The pooled correlation was r = −0.42 (95% CI − 0.52 to −0.31), a moderate inverse association: samples reporting higher resilience-related resources also reported lower traumatic stress. Heterogeneity was substantial (I^2^ = 79.1%), reflecting differences in how resources and outcomes were defined across studies (psychological resilience versus hardiness versus effective coping, and PTSD symptoms versus secondary traumatic stress versus rumination). Excluding studies rated at moderate-to-high risk of bias left the estimate unchanged, since none of the four received that rating. Extending the synthesis beyond health professionals by adding the coefficient reported by Bakirci et al. (2024) among firefighters engaged in urban search and rescue (PCL-5 with the Brief Resilience Scale, r = −0.436) moved the pooled estimate only from r = −0.420 to r = −0.423 (95% CI − 0.504 to −0.335), with I^2^ = 72.3%; because this rests on the addition of a single effect, it is reported as a limited rather than a decisive check. Figure 3 presents the four contributing coefficients and the pooled result.

**Figure 3:**
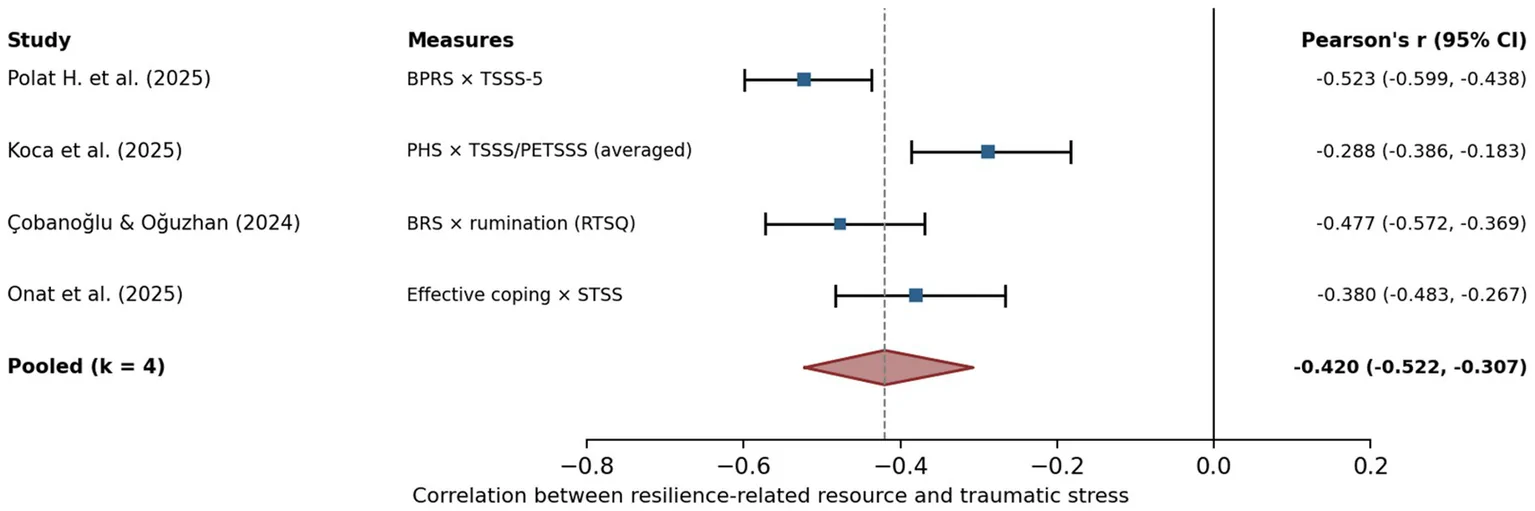
Forest plot of pooled associations (Pearson’s r) between resilience or hardiness measures and traumatic stress outcomes among health professionals.

### Multivariate and structural model findings

Four studies went beyond bivariate analyses by using multivariate statistical models or structural equation modeling (SEM) to examine the mechanisms linking resilience, coping, and traumatic stress outcomes in health workers (Table 4). These studies collectively tested direct and indirect pathways—for example, whether resilience effects were mediated by coping strategies or influenced by external factors—in order to better understand how resilience processes operate in the context of disaster-related trauma.

**Table 4:** Structural equation and multivariate models examining mechanisms of traumatic stress.

Author (Year)	Population	Model type	Key predictors	Outcome(s)	Main pathways/Effects
Koca et al. (2025)	Survivor HCWs	SEM (mediation)	Hardiness (PHS), Coping (CRI)	Traumatic stress (TSSS, PETSSS)	Hardiness → Coping (β = 0.52); Hardiness → ↓ Stress (β ≈ −0.19); Coping → ↓ Stress (β ≈ −0.13); partial mediation
Kiymis and Yuce (2025)	Survivor HCWs	SEM	Trauma exposure, Disaster resilience	PTSD, Depression, Sleep disturbance	Resilience → ↓ PTSD (β ≈ −0.28); ↓ Depression (β ≈ −0.32); ↑ Sleep disturbance (β ≈ +0.69)
Polat I. et al. (2025)	Deployed HCPs	Multiple regression	Fatigue, unmet needs, workload	PTSD symptoms (IES-R)	Fatigue and unmet basic needs predicted higher PTSD symptoms
Onat et al. (2025)	Vicarious exposure HCWs	Regression/correlational	Coping styles	Secondary traumatic stress	Ineffective coping → ↑ STS; effective coping → ↓ STS

In a large sample of health workers who were earthquake survivors, Koca et al. (2025) applied an SEM mediation model to investigate hardiness and coping. They reported that psychological hardiness had both direct and indirect inverse associations with post-traumatic stress severity within the fitted model. Health workers with higher hardiness tended to utilize more adaptive coping strategies (Koca et al. reported a strong path coefficient, β ≈ 0.52, for hardiness predicting effective coping). Those adaptive coping strategies in turn predicted lower traumatic stress scores (β ≈ −0.13). Even after accounting for coping, hardiness also maintained a direct negative link to stress symptoms (direct effect β ≈ −0.19). This partial mediation indicates that within the fitted model hardiness—a resilient personality trait—was associated with lower distress both indirectly, through its association with coping behaviour, and through other direct psychological pathways (e.g., better emotion regulation or optimism) that independently lessen trauma symptoms.

Kiymis and Yuce (2025) similarly used a structural equation model to examine how disaster resilience related to mental health outcomes in survivor health workers. In their model, higher resilience was associated with significantly lower PTSD symptoms, as well as lower depression symptoms. However, an unexpected finding was that resilience was positively associated with sleep disturbance (they observed a relatively large standardized coefficient, β ≈ +0.69, linking resilience to increased sleep problems). This counterintuitive result is reported here as an isolated and unreplicated finding. One speculative reading is that certain dimensions of being “resilient” or highly dedicated in a disaster context—for instance, persistent hyper-vigilance, taking on extra duties, or difficulty unwinding—might come at the cost of disrupted sleep, even while psychological trauma symptoms are kept in check. In other words, resilience in this context may have mixed outcomes, improving some aspects of mental health but potentially exacerbating fatigue or physiological arousal that interferes with sleep.

Among studies of deployed responders and indirectly exposed staff, the analyses highlighted the role of situational stressors and coping styles. Polat I. et al. (2025) conducted a multivariate regression focusing on field hospital personnel deployed after the earthquakes. They identified fatigue and unmet basic needs (such as lack of food, water, or rest in the field) as significant predictors of higher PTSD symptom levels. In their findings, responders who experienced greater physical exhaustion and who lacked adequate accommodations or supplies showed elevated traumatic stress scores, even after controlling for other factors. This underscores how organizational and logistic factors—not just personal resilience—can strongly influence mental health outcomes during disaster response. Similarly, Onat et al. (2025) examined predictors of secondary traumatic stress in health workers and found that coping style was central: those who habitually engaged in ineffective coping strategies (e.g., denial, self-blame, or behavioral disengagement) had significantly higher secondary stress, whereas those using effective coping (e.g., active problem-solving, seeking support) had lower secondary stress levels. This reinforces the quantitative correlation findings that coping strategies can either amplify or alleviate the psychological impact of secondary trauma.

In summary, these multivariate and structural model findings provide a more nuanced understanding of how resilience operates in disaster-exposed health workers. The evidence suggests that resilience-related traits (like hardiness or general disaster resilience) influence outcomes via multiple pathways: through individual-level processes such as coping strategy selection and emotion regulation, and through interactions with the contextual factors of the work environment (like workload, fatigue, and resource availability). Due to differences in model design and variables, it was not feasible to statistically pool these complex models; instead, we synthesize them narratively. Collectively, however, the pattern is that both personal resilience and external supports (or stressors) jointly determine the level of trauma-related distress. These quantitative model results will be integrated with the qualitative findings to form a comprehensive picture of health worker resilience and mental health in this disaster context.

### Qualitative thematic synthesis

The five qualitative studies provided rich, contextual insights into the psychological impact of the earthquakes and the resilience strategies employed by health professionals. Across these studies, participants consistently described experiencing intense emotional and psychological distress in the aftermath of the Kahramanmaraş earthquakes. Health professionals who survived the disaster or responded to it reported acute fear and feelings of helplessness during the earthquake and its immediate aftermath, followed by intrusive recollections of the traumatic events. Many described profound emotional exhaustion and burnout, and sleep disturbances were commonly mentioned, with some participants reporting nightmares or insomnia related to their experiences. Notably, these distress reactions were observed both among health professionals who directly survived the earthquake and among those deployed to assist, indicating the broad psychological toll of working in a disaster environment. The two reports added during revision reinforced this pattern from complementary vantage points: Mert and Köksal (2025) interviewed volunteer nurses within days of returning from deployment, while Deniz Doğan et al. (2024) interviewed nurses resident in affected provinces who were caring for survivors, and both described the same constellation of exhaustion, fear of aftershocks and moral strain.

A prominent theme that emerged was secondary traumatization among health professionals, particularly nurses and emergency personnel who cared for severely affected survivors. Participants recounted that repeatedly listening to patients’ harrowing stories of loss and suffering took an emotional toll. They often felt emotionally overwhelmed and had difficulty mentally detaching from the trauma of others. For instance, some described going home after shifts yet being unable to stop thinking about the patients and tragedies they had witnessed, leading to persistent worry and guilt. This qualitative theme of secondary or vicarious trauma closely mirrors the elevated secondary traumatic stress scores documented in the quantitative studies of indirect exposure (eg., Onat et al., 2025), thereby reinforcing the validity of those self-reported symptoms with real-world narratives.

Despite the high levels of distress, health workers also described a variety of coping mechanisms and resilience strategies that helped them manage their psychological load. One widely cited coping strategy was meaning-making and spirituality. Many participants talked about drawing strength from their faith or spiritual beliefs—for example, engaging in prayer, reciting religious texts, or believing that surviving the disaster had a larger purpose. Such spiritual coping provided comfort and a sense of hope or acceptance amidst chaos. These accounts align with the quantitative findings that highlighted the use of religious coping and positive reappraisal as effective strategies (e.g., Dağlar et al., 2025, noted a correlation between religious coping and positive cognitive reframing). In essence, finding meaning through faith or reframing the disaster in a more hopeful light was a key resilience mechanism described across multiple studies.

Social support was another resource discussed consistently. Participants emphasized the importance of peer and team support during the disaster response. Simply sharing their experiences and feelings with colleagues, debriefing after difficult shifts, and providing mutual encouragement helped them feel less alone and more capable of coping. A sense of camaraderie and “we’re in this together” solidarity among health workers emerged as a buffer against stress. Several interviewees mentioned that knowing their colleagues understood what they were going through, and being able to lean on each other emotionally, was vital for sustaining their mental health during the crisis. This qualitative emphasis on collegial support echoes findings in the quantitative literature on the value of social support in trauma settings.

In contrast, some participants described using psychological distancing techniques as a way to cope with the overwhelming situation. This included deliberate avoidance of reminders of the disaster—for instance, avoiding watching news coverage of the earthquake or steering clear of devastated areas when possible—and emotional suppression while on duty. Health workers felt they had to “switch off” their personal feelings temporarily in order to maintain professional performance and make urgent decisions. While these avoidance strategies were often deemed necessary in the short term (enabling them to function during acute response), a few participants acknowledged that there were emotional costs to prolonged suppression. Some reported that once the immediate work settled, they experienced a delayed emotional response or a sense of numbness, suggesting that unaddressed emotions eventually resurfaced. Thus, avoidance had a dual character: it was a coping method that provided relief in the moment but was not sustainable as a long-term strategy for many individuals.

These qualitative findings yielded several overarching themes regarding psychological impact (e.g., acute distress, secondary trauma) and resilience strategies (e.g., spirituality/meaning-making, social support, and controlled distancing), as well as insights into organizational factors (like systemic challenges or supports). For an organized summary of the core themes and illustrative examples from the qualitative data (see Table 5).

**Table 5:** Qualitative themes on psychological impact and resilience strategies among health professionals.

Theme domain	Core theme	Description	Representative evidence (studies)
Psychological impact	Acute and persistent distress	Fear, helplessness, intrusive memories, emotional numbing and sleep disruption following exposure	Emirza; Köksal; Uysal; Mert & Köksal; Deniz Doğan
Psychological impact	Secondary traumatization	Distress arising from caring for severely affected survivors, even without direct earthquake exposure	Emirza; Onat; Deniz Doğan
Resilience strategies	Meaning-making and spirituality	Prayer, faith and existential reframing used to tolerate uncertainty and loss	Emirza; Dağlar; Köksal
Resilience strategies	Social and peer support	Emotional sharing with colleagues, teamwork and perceived solidarity	Emirza; Köksal; Uysal; Mert & Köksal
Resilience strategies	Psychological distancing	Avoidance of earthquake-related media and suppression of emotions to maintain functioning	Emirza; Uysal; Köksal
Organizational factors	System-level barriers	Inadequate accommodation, fatigue, supply shortages, coordination problems	Polat I.; Uysal; Köksal; Mert & Köksal; Deniz Doğan
Organizational factors	Facilitators of coping	Feeling valued, access to rest, psychological support availability	Köksal; Uysal; Mert & Köksal

### Integrated mixed-method findings

Integrating the quantitative and qualitative evidence reveals a strongly convergent story about how health workers were psychologically affected by the earthquakes and what helped them cope. The quantitative results demonstrated that individuals with greater resilience—whether defined as general psychological resilience, hardiness, or effective coping skills—tended to experience lower levels of PTSD symptoms and stress. The qualitative narratives put these statistical findings into context by illustrating how resilience manifested in practice: health workers described using personal and collective coping strategies (like finding spiritual meaning, cognitively reframing the situation, relying on teamwork and peer support) that correspond to the constructs measured in the surveys. In other words, the qualitative data show the human behaviors behind the numbers, confirming that those who fared better psychologically often did so by actively drawing on internal strengths and external support systems.

Conversely, both data strands identified organizational and system-level factors as significant determinants of psychological outcomes. Several quantitative studies (e.g., Polat I. et al., 2025) found that practical stressors—such as extreme fatigue, lack of rest or sleep, inadequate accommodation, and unmet basic needs during deployment—were associated with worse PTSD or stress outcomes. The qualitative accounts vividly described these same issues: health workers spoke of exhaustion from long hours, difficulties in obtaining food or shelter in the field, and frustration with coordination problems or resource shortages. Participants noted that insufficient support and logistical challenges not only impeded their work but also amplified their emotional stress. The mixed-method synthesis thus underlines that resilience is not just an individual trait; it is also heavily influenced by the context in which one is operating. Even the most resilient person can be stretched thin if they are sleep-deprived and lacking support, whereas a supportive work environment can bolster individuals who might otherwise struggle.

Importantly, the qualitative insights helped explain some of the heterogeneity observed in the quantitative findings. For example, differences in PTSD prevalence between studies of direct survivors vs. deployed responders align with narratives that direct personal losses and chaos (as faced by survivors) produce higher distress than the challenges faced by those who came to help later. The timing of assessments also matters: qualitative reflections suggested that many health workers initially suppressed emotions to get through the crisis, with some psychological symptoms emerging only after some time had passed—which could contribute to why studies at different post-disaster intervals found varying prevalence rates. Additionally, the presence or absence of strong organizational support structures (e.g., access to psychological support, adequate rest periods, effective leadership) was frequently mentioned qualitatively and likely contributed to variations in outcomes across settings. By triangulating the evidence, it becomes clear that the psychological impact on health workers following a large-scale disaster is shaped by a dynamic interplay of factors: the intensity of trauma exposure (direct vs. indirect), the individual’s resilience and coping capacities, and the broader organizational context and resources available to them. Integrating quantitative and qualitative findings provides a richer understanding that while personal resilience was strongly associated with better mental health, external working conditions and support were associated at least as strongly with the long-term psychological toll on health professionals in disaster settings.

## Discussion

### PTSD prevalence and exposure differences

Our review found a high burden of trauma-related psychological difficulty among health professionals following the Kahramanmaraş earthquakes, but the distribution of that burden across exposure contexts is less clearly patterned than the early literature suggests. Among health professionals who were direct survivors, prevalence estimates for probable PTSD clustered around 40–50% several months after the disaster: Aksoy et al. (2025) reported that 47.7% of surveyed staff met the IES-R screening cut-off, and Polat H. et al. (2025) reported 39.6% on the TSSS-5. That roughly two in five directly affected providers screened positive some months after the event is a substantial finding, and it is consistent with estimates from general survivor samples in the same disaster, indicating that professional status was not accompanied by a lower prevalence.

We also noted that health workers with indirect or vicarious exposure—for instance, those working with survivors outside the immediate disaster zone—experienced considerable traumatic stress, albeit typically in subclinical or moderate ranges. Onat et al. (2025) documented elevated Secondary Traumatic Stress Scale scores in personnel caring for evacuees, indicating that repeatedly hearing patients’ harrowing stories and witnessing their suffering induced significant distress. Qualitative accounts vividly corroborated this phenomenon of secondary trauma: nurses and emergency staff described feeling emotionally overwhelmed by patients’ narratives of loss, to the point of being unable to “switch off” thoughts of their patients even after their shifts ended. In other words, simply working with disaster survivors—even without being a survivor oneself—was enough to generate intrusive images, empathic pain, and guilt among many health workers, a finding consistent with the broader literature on vicarious traumatization in caregiving professions.

The position of deployed personnel is more equivocal. Polat I. et al. (2025) found 8.1% probable PTSD among field hospital staff at one to two months using IES-R ≥ 33, whereas Satılmış et al. (2024) found 37.9% among health-care workers assigned to the earthquake zone at approximately two months using PCL-5 ≥ 47—a more than fourfold difference between two studies of the same exposure stratum, assessed at a similar interval. Pooling them gives an estimate whose confidence interval spans 3.5 to 61.8%. The available evidence therefore does not support the inference that deployment carries a categorically lower risk than direct survivorship. Measurement decisions are one plausible contributor to this dispersion: studies of survivor populations in this literature have tended to use instruments and thresholds with higher sensitivity, while studies of deployed responders have more often used stringent cut-offs such as PCL-5 ≥ 47, a threshold substantially above the value of 31–33 now commonly recommended for the PCL-5 in screening contexts. We are, however, cautious about pressing this explanation further than the data allow. The two studies applying PCL-5 ≥ 47 agreed almost exactly (39.0%, I^2^ = 0.0%), but the two applying IES-R ≥ 33 diverged as widely as any pair in the dataset (8.1 and 47.7%) despite sharing both instrument and threshold. Measurement approach alone therefore cannot account for the heterogeneity, and with five studies spread across three instruments, three exposure categories and assessment intervals from one to eleven months, instrument, exposure, timing, sampling frame and setting cannot be disentangled. We present measurement heterogeneity as one plausible contributor among several rather than as the primary explanation, and we would caution readers against reading any prevalence contrast in this literature as a contrast in risk until primary studies converge on common instruments.

This has a practical implication for how this literature should be read and extended. Comparisons of PTSD prevalence across occupational or exposure groups after the Kahramanmaraş earthquakes will remain difficult to interpret until primary studies converge on common instruments and report symptom distributions rather than dichotomised rates alone. We would encourage future primary studies to report full score distributions, so that prevalence at any threshold can be recovered by subsequent syntheses, and we note that our own pooled estimates should be revised as such data become available. Heterogeneity in these findings also reflects timing: some surveys were conducted one to two months post-disaster, capturing acute stress reactions, whereas others took place nearly a year later. Our qualitative data offer an explanatory nuance, in that many health professionals described suppressing their emotions during the emergency phase in order to function, only to experience a delayed onset of symptoms once the immediate crisis passed.

The association between resilience-related resources and traumatic stress was more stable across the analyses we were able to run than the prevalence estimate was. Adding a coefficient from firefighters engaged in urban search and rescue moved the pooled estimate from r = −0.420 to r = −0.423 and slightly reduced heterogeneity. This is a limited check, resting on a single added effect from four contributing samples, and it should not be read as establishing that the association generalises across occupational groups. What can reasonably be said is that the direction of association was uniform across every sample and every operationalisation we examined, whereas the prevalence estimate varied by a factor of nearly six across studies of nominally similar populations. Direction appears to be the more transportable feature of this evidence base; magnitude, and prevalence in particular, does not.

### Resilience and hardiness in relation to traumatic stress

Against this backdrop of elevated trauma exposure, a consistent finding from our synthesis concerns the association between resilience-related personal resources and outcomes. Across virtually all quantitative studies, higher levels of resilience, hardiness or effective coping were associated with lower levels of PTSD symptoms and psychological distress among health workers. The direction of this association was uniform—every study that measured resilience or a similar construct found it inversely linked with distress—even if the magnitude varied. Reported coefficients between resilience-related measures and trauma symptoms ranged from small-to-moderate (around r = −0.28) to large (around r = −0.52). Our meta-analysis entered one coefficient from each of four independent samples, following the selection rule set out in the Methods, and yielded a pooled correlation of r = −0.42 (95% CI − 0.52 to −0.31; I^2^ = 79.1%). Because the contributing studies were cross-sectional and reported continuous associations rather than risk estimates, this coefficient should not be read as an odds ratio or as an estimate of risk reduction; it describes the degree to which the two sets of scores covaried within the same measurement occasion. The finding is consistent with a broad body of trauma research reporting that resilience accompanies lower rates of post-traumatic stress, though that literature is also predominantly cross-sectional and shares the same interpretive limits.

The strongest single association came from Polat H. et al. (2025), who reported r = −0.523 between brief-resilience scores and TSSS-5 symptom severity among survivor health personnel: within that sample, participants reporting higher resilience also reported markedly lower symptom scores at the same time point. Koca et al. (2025) reported more moderate coefficients between hardiness—a construct reflecting commitment, control and challenge in stressful situations—and traumatic stress severity (r = −0.281 and −0.294 on two outcome scales), indicating that participants scoring higher on hardiness reported less severe stress reactions. We note that neither study permits a statement about how much lower symptoms would be were resilience raised, and we have avoided such formulations. Resilience-related measures were also associated with positive outcomes: Kınık et al. (2024) found that responders scoring higher on hardiness also reported greater compassion satisfaction (r = +0.57), suggesting that in some samples higher resilience scores co-occurred not only with less distress but with a greater reported sense of meaning and efficacy in the disaster response role. That coefficient was not entered into the pooled model, since compassion satisfaction is a positive occupational construct rather than a traumatic stress outcome.

The consistency of the direction of these findings is notable. Despite differences in how studies defined resilience—some used general resilience scales, others focused on specific facets such as optimism or coping styles—all pointed the same way: higher resilience-related scores accompanied lower symptom scores. Even maladaptive processes such as rumination were inversely related to resilience; Çobanoğlu and Oğuzhan (2024) found that nurses reporting higher resilience also reported less rumination about the trauma (r = −0.48). What these data cannot establish is the direction of influence. A correlation of this magnitude is equally compatible with resilience buffering distress, with distress eroding self-reported resilience, and with both being driven by a third factor such as cumulative exposure or organisational support. Longitudinal designs are required before the clinical inference that raising resilience would lower post-disaster psychopathology can be drawn from this literature.

That said, our review also uncovered nuances in how resilience operates. The structural equation modeling (SEM) studies allowed a closer look at the mechanisms. Koca et al. (2025) fitted a model in which the association between resilience and distress was partly indirect, running through coping strategies. In their model, hardy individuals were more likely to engage in adaptive coping (e.g., problem-solving, positive reframing), which was in turn associated with lower PTSD symptoms. Even after accounting for coping, hardiness retained a residual direct association with lower distress within the model. Because the model was fitted to cross-sectional data, these pathways describe patterns of covariation rather than demonstrated mechanisms. This suggests that interventions can potentially target both the enhancement of personal resilience and the promotion of healthy coping behaviors as complementary strategies—a point we will return to when discussing implications.

A particularly intriguing finding came from Kiymis and Yuce (2025), who reported an unexpected positive association between resilience and sleep disturbance in their SEM analysis. While higher resilience was linked to lower PTSD and depression (as expected), it was also linked to increased insomnia and poor sleep quality (β ≈ +0.69). This initially seems counterintuitive—we would assume a resilient person sleeps better, not worse. We report this as an exploratory and unreplicated observation from a single structural model, and the interpretation that follows is offered as a hypothesis to be tested rather than as an explanation supported by the present evidence. With that caveat, the qualitative context offers one possible reading. Many health workers described a relentless sense of duty and hyper-vigilance: the most “resilient” individuals in a professional sense were often those pushing themselves hardest, working extra shifts, staying alert for aftershocks or new patients, and struggling to “wind down” at night. It appears that resilience, in the form of strong dedication and perseverance, came at a cost: these workers might suppress their anxiety successfully during the day, only to have it manifest as physiological arousal and sleeplessness at night. In other words, being very mission-focused and hardy helped them keep PTSD symptoms at bay and continue functioning, but it also meant they had difficulty relaxing or showing vulnerability, leading to disturbed sleep. This finding is a valuable reminder that resilience is not a panacea and can have trade-offs. We interpret this in line with concepts of “resilience fatigue”—the idea that maintaining a brave front and high alertness through chronic stress can wear on the body in subtler ways (like disrupting circadian rhythms). From a support standpoint, it underlines that even those coping “well” on the surface may need help with basic self-care such as sleep and rest. It also cautions against an overly simplistic view of resilience; effective support programs should encourage healthy rest and recovery, not just continuous toughness.

### Coping strategies: quantitative findings and qualitative narratives

Quantitative studies that measured coping consistently found that adaptive coping strategies were associated with better psychological outcomes, whereas maladaptive coping was associated with worse outcomes. For instance, Onat et al. (2025) reported that health workers who utilized effective coping styles (e.g., active problem-solving, seeking emotional support, positive appraisal) had significantly lower secondary traumatic stress (r ≈ −0.38). In contrast, those who frequently relied on ineffective coping (e.g., denial, behavioral disengagement, self-blame) showed higher secondary stress (r ≈ +0.48). This paints a clear picture that coping style was associated with the severity of psychological impact in both directions. Similarly, Aksoy et al. (2025) and others identified positive reappraisal—reframing the situation to find some meaning or positive angle—as a recurring element in resilient coping. Positive reappraisal was often linked with lower PTSD symptoms, suggesting that the ability to cognitively process the event in a constructive way helped health workers avoid getting stuck in despair.

These statistical patterns come alive in the qualitative narratives from our synthesis. Health workers repeatedly mentioned specific coping methods that either sustained them or, in some cases, proved insufficient. A dominant theme was meaning-making and spirituality as coping mechanisms. Many participants described drawing strength from faith, prayer, or the belief that there was a “reason” they survived and a higher purpose to their work. For example, nurses in one interview study spoke of how praying or reciting religious texts gave them comfort and a sense of guidance amid chaos. This qualitative theme directly aligns with our quantitative findings—notably, Dağlar et al. (2025) found that religious coping was significantly correlated with positive cognitive reframing (r = 0.40). In practice, this means that those who coped by turning to spiritual beliefs were often the same individuals actively trying to see the disaster in a survivable or meaningful light (“perhaps this is a test,” “we have become closer to our community,” etc.). The convergence of evidence here strengthens confidence in the importance of existential or spiritual coping for this population. It provides a culturally relevant insight too: in Turkish society (as in many others), faith can be a vital source of resilience, and interventions that incorporate or at least respect this facet (such as partnering mental health services with spiritual counselors) might amplify engagement.

Another key coping resource was social support. Quantitative disaster research often reports social support as among the factors most consistently associated with lower PTSD risk, and our review is no exception. Although not all our included quantitative studies measured social support directly, it is inherently embedded in many resilience scales and coping inventories (e.g., seeking support is a coping subscale). Moreover, in Kınık et al. (2024), the concept of compassion satisfaction can partly be seen as deriving from collegial support and teamwork (finding satisfaction in helping others). The qualitative findings underscored that health workers leaned heavily on each other: peer support and teamwork emerged as critical buffers against stress. Participants described how debriefing with colleagues after exhausting shifts, sharing emotions with teammates who “understand what I’ve been through,” and rallying together to solve problems helped them not to feel alone. A sense of camaraderie—the feeling that “we were all in this together”—was frequently mentioned as something that kept them going in dark moments. This meshes with disaster literature emphasizing that group cohesion and mutual support can foster collective resilience in emergency settings. From a quantitative angle, one might expect that in settings where such support was strong, overall PTSD rates would be lower (even if our review did not have a formal metric for it, the qualitative evidence suggests a protective effect). Indeed, the absence of social support is a known risk factor; the general survivor studies of the 2023 earthquakes identified low social support as predictive of worse mental health. For health workers, peer support is not only emotional but also instrumental—covering duties for one another, sharing supplies, etc., which can directly reduce stressors. Thus, our integrated findings confirm that encouraging strong team dynamics and peer communication is an essential part of helping providers cope.

In contrast, avoidant coping was a double-edged sword as described by participants. On one hand, nearly all health workers acknowledged engaging in some psychological distancing to survive the immediate crisis: emotional numbing, suppressing feelings, or avoiding reminders of the earthquake were commonly reported short-term tactics. For example, some deliberately avoided watching news about the disaster or refused to visit the hardest-hit neighborhoods on rare off-days. Many spoke of “switching off” their personal emotions while on duty so they could function professionally. These behaviors correspond to what the quantitative literature labels as maladaptive or ineffective coping—essentially forms of denial or avoidance. In the acute phase, such coping can be adaptive in that it allows one to push through extreme situations. Indeed, it is understandable that a surgeon in an overwhelmed field hospital might compartmentalize feelings of grief in order to keep operating. However, participants noted that prolonged reliance on avoidance took a toll. Once the urgency subsided, those suppressed emotions often resurfaced, sometimes in the form of numbness or delayed anxiety. A few health workers recounted that after weeks of working nonstop, when they finally had a quiet moment, they felt an unexpected wave of anguish—a sign that avoidance had merely postponed the reckoning. This resonates with Onat et al.’s quantitative finding: those who habitually used avoidance and other ineffective strategies ultimately had higher trauma scores. In sum, avoidance provided temporary relief but was not sustainable as a long-term coping strategy for many. The practical lesson is that while some degree of psychological distancing is a natural acute stress response, health workers need help transitioning to more proactive coping once the immediate crisis is over, lest unaddressed trauma accumulate.

In integrating these findings, a coherent picture emerges: the health workers who fared better mentally after the earthquakes were typically those who (whether by personal inclination or external support) engaged in adaptive coping—finding meaning, leaning on colleagues, confronting challenges with problem-solving—whereas those who struggled more tended to be isolated, overwhelmed, or reliant on short-term emotional suppression. This does not imply blame on individuals; rather, it highlights leverage points for interventions. Training and resources could be provided to encourage effective coping (for example, workshops on stress management techniques, peer support groups, or mentoring systems that foster shared coping). Our review’s mixed-methods design strengthens confidence in these conclusions, as we see the same themes reverberate in both statistics and stories: resilience and coping skills matter greatly, and they manifest in very human ways on the ground, from praying at daybreak to hugging a coworker after a hard shift.

### Organizational and system-level influences

One of the clearest messages from this review is that the mental health of disaster-exposed health workers is not just about individual resilience or personal coping capacity—the organizational and systemic context in which they work is equally crucial. Several of the included studies explicitly examined how working conditions and support (or lack thereof) affected outcomes, and the qualitative findings resoundingly echoed these factors.

On the quantitative side, Polat I. et al. (2025) provided striking evidence that operational stressors predicted PTSD symptoms among healthcare responders. In their multivariate regression, two factors stood out: extreme fatigue and unmet basic needs during deployment were significantly associated with higher PTSD scores. In practical terms, responders who were physically exhausted from working endless hours and those who lacked access to adequate food, water, shelter, or rest had markedly worse mental health. This finding might seem intuitive, but it has important implications—it suggests that even if two individuals have similar personal resilience, the one forced to operate without sleep or proper meals will be at far greater risk of psychological breakdown. Another quantitative study noted that heavy workload and prior burnout (common issues in Turkish emergency departments even before the quakes) likely predisposed staff to PTSD when the disaster struck. In other words, systemic pressures that predate the disaster (like chronic understaffing or high violence against hospital staff) set the stage such that when a calamity occurred, those underlying strains amplified the impact.

The qualitative data provide a firsthand look at these system-level problems. Health workers spoke frequently about logistical and organizational challenges that exacerbated their stress. Common themes included: lack of proper accommodations (some responders slept in cars or tents for weeks), shortages of medical supplies and medications, chaotic coordination and communication breakdowns, and exceedingly long shifts with no relief. For example, one focus group of emergency nurses (Uysal et al., 2025) described how the hospital’s collapse and ad-hoc field clinics meant they were often working in improvised conditions without sufficient equipment or lighting, all while dealing with a never-ending flow of patients. Such conditions bred frustration and feelings of helplessness, which compounded their emotional toll. Participants also mentioned the absence of formal psychosocial support in the early weeks—there were few if any counselors or mental health professionals available for the staff, and no organized debriefings. Many only received psychological help much later, if at all, by which time their symptoms had hardened. On the flip side, some positive outliers were noted: a few health workers recalled a particularly supportive supervisor or a well-organized relief team that rotated staff out regularly for rest, and they believed these factors protected their mental health. These anecdotes mirror the finding in our integration that contexts with strong support structures tended to see better outcomes.

Bringing these pieces together, our mixed evidence underscores that resilience is as much collective and systemic as it is individual. A resilient health system (or hospital) can bolster the resilience of its workers, whereas a failing system can defeat even otherwise resilient individuals. Even the most dedicated nurse can be “brought to their knees,” as one participant phrased it, by relentless 20-h shifts and the moral distress of having inadequate resources to help patients. Conversely, a moderately stressed provider might cope fine if given proper rest, meals, and psychological support. This interplay was exemplified starkly in the data: exhaustion and burnout emerged as key mediators. Several responders noted that physical fatigue often triggered their emotional breakdowns—“when I got too tired, I’d start crying over little things,” one doctor admitted, illustrating how the body and mind are linked in trauma. Unmet basic needs (like not having a safe place to sleep) kept the nervous system in a state of threat, preventing recovery. Thus, addressing these needs is not just a humane logistic issue but a mental health intervention in itself.

Polat I. et al.’s (2025) findings also highlight the concept of moral injury—responders felt the system’s shortcomings (supplies not arriving, etc.) were a betrayal that left them unable to fulfill their duty, contributing to trauma. Indeed, lack of organizational support was often internalized by workers as a sense that they were “hung out to dry,” which can be deeply demoralizing. On the other hand, when organizations did provide support (e.g., arranging counselling or publicly recognizing staff efforts), it boosted morale and coping. One qualitative theme we identified was “facilitators of coping” at the system level, such as feeling valued by leadership, having access to rest periods, and clear communication channels. These might seem like mundane administrative matters, but in a disaster scenario they become pivotal for mental health. Our integrated analysis concluded that ensuring such supports is associated with better psychological outcomes among health workers.

### Implications for support and future preparedness

This mixed-methods review carries several important implications for mental health support interventions and disaster preparedness planning aimed at healthcare workers. First and foremost, the magnitude of PTSD and distress observed—with roughly 40–50% of directly exposed health workers affected—signals an urgent need for proactive and sustained psychosocial support services for this group. Just as survivors in the community receive counseling and resources, frontline providers should also be offered screening, monitoring, and therapeutic services in the aftermath of mass trauma. Hospital administrators and public health authorities should implement routine mental health screenings for staff following a disaster, as recommended by Çifçi and Kılınç (2024), to identify those at risk (our review suggests women, nurses, and those with personal losses may be priority groups). Early intervention—for example, stress debriefings, peer support sessions, and referral to professional counseling—could mitigate the development of chronic PTSD by addressing symptoms in their initial stages.

Our findings on resilience and coping indicate that building these capacities should be a central focus of interventions. Training programs or workshops for health workers in disaster-prone regions might include modules on effective coping strategies (e.g., problem-solving techniques, breathing and mindfulness exercises for acute stress, strategies for positive reframing). There is evidence from broader trauma research that resilience can be cultivated through skill-building and that even simple practices (like encouraging workers to reflect on success stories or gratitude amid crisis) can foster a more resilient mindset. Organizations can facilitate peer support networks or buddy systems so that health workers naturally have someone to talk to and lean on during and after events. The clear protective value of social support seen in our review suggests that anything which strengthens collegial bonds—team-building activities, structured debriefs, or peer mentorship—will pay dividends in mental health outcomes. Indeed, creating a culture where asking for help is encouraged and emotional openness is not stigmatized is key. Traditionally, many medical professionals feel compelled to appear tough and composed (“professional resilience”), which can discourage them from seeking help. Leadership should counteract this by openly acknowledging stress and modeling help-seeking behaviors (e.g., department heads partaking in counseling themselves, or bringing in counsellors to rounds).

On the organizational side, the takeaway is that disaster preparedness plans must integrate workforce support as a pillar. Just as hospitals stockpile supplies and simulate mass-casualty responses, they should also have protocols for staff care. This could include ensuring rotating shifts (no provider should work indefinitely without relief, even if it means bringing in external volunteers to cover), establishing rest areas with food and sleeping quarters at emergency sites, and deploying mental health professionals as part of the disaster response team to specifically tend to rescuers (psychological first aid units for staff). The data on fatigue and unmet needs correlating with PTSD make it evident that taking care of basic needs is a form of psychological prevention. International guidelines on emergency psychosocial support emphasize providing practical help and physical comfort as the first steps in trauma mitigation. Our review validates that for health workers: something as simple as a hot meal and a few hours of sleep may matter for mental health.

Additionally, communication and leadership emerged as influential. Training hospital and emergency response leaders in trauma-informed leadership could be beneficial. Leaders who keep staff informed, express empathy, and actively solicit feedback can reduce confusion and empower workers, which helps morale. Conversely, poor coordination and perceived mismanagement fueled distress and moral injury in some accounts. Thus, improving disaster logistics (clear incident command structures, backup communication systems if phones fail, etc.) is not only operationally smart but also psychologically beneficial for staff who otherwise feel “abandoned” or in chaos.

Given the heterogeneity of responses and the possibility of delayed onset of issues, long-term follow-up is necessary. Mental health support should not cease a few weeks after the disaster; many health workers will only process their trauma months later once the adrenaline subsides. Instituting follow-up checks at 6 months, 1 year, and even beyond (with referrals for those still struggling) would ensure that late-arising cases of PTSD or depression are caught and treated. This is in line with recommendations from prior disaster studies that emphasize sustained psychosocial services throughout the recovery phase.

Our findings regarding the sleep disturbance paradox also suggest interventions should address health behaviors. Encouraging and possibly mandating rest periods, educating staff about sleep hygiene, and, if needed, providing medical management for sleep (short-term use of sleep aids or relaxation techniques) could prevent the resilient-yet-sleepless phenomenon from eroding health. Since hardiness sometimes led people to overextend themselves, managers might need to enforce time off for those who insist on working nonstop (to protect them from burnout).

Finally, the multifaceted nature of resilience highlighted by this review implies that support strategies must operate on multiple levels. At the individual level, bolstering psychological resources (through counseling, skills training, stress management tools) is vital. At the social level, support networks and team cohesion were associated with better outcomes. And at the structural level, improving working conditions, ensuring safety, and meeting basic needs are fundamental. Disaster response plans should be comprehensive in addressing all three levels. For example, a program for health workers post-earthquake might combine personal counseling (individual), peer support meetings (social), and policy changes like shorter shifts and scheduled rotations out of the disaster zone (structural). Such an integrated approach recognizes that personal resilience will falter if structural supports are absent, and conversely that even a well-organized system needs individuals who are mentally prepared and supported to function within it.

### Limitations

Several limitations qualify these findings. The evidence base is small and, for the prevalence synthesis, very small: only five studies reported dichotomous probable-PTSD data, distributed across three instruments and three exposure categories, so that instrument cannot be statistically separated from exposure and the subgroup estimates must be read as descriptive. Heterogeneity was high throughout (I^2^ = 86.2% for prevalence and 79.1% for correlations), and the pooled prevalence should be understood as a summary of a dispersed distribution rather than as a stable population parameter. The variation in thresholds used to define probable PTSD—IES-R ≥ 33, TSSS-5 ≥ 25 and PCL-5 ≥ 47—is wide enough that the same sample would yield materially different prevalence estimates depending on which instrument had been administered. We are nonetheless unable to attribute the observed heterogeneity to any single source: instrument, threshold, exposure context, assessment interval, sampling frame and setting vary together across these five studies, and the two studies sharing both instrument and threshold were among the most discrepant in the dataset. The subgroup and leave-one-out analyses should therefore be read as descriptive rather than as identifying a cause of dispersion.

The search was restricted to English and Turkish, and although nine sources were searched and citation chasing was performed in both directions, unindexed Turkish theses, institutional reports and other grey literature may not have been fully captured. The search closed on 30 September 2025; studies published subsequently, including reports of healthcare-worker outcomes at the one-year and later anniversaries, are not represented and will need to be incorporated by future updates. One study assessed at full text during revision, Sert et al. (2024), was excluded on reflection because its participants were not present in the affected region and no clinical care of earthquake survivors was documented, placing them outside the predefined population; it is recorded with this reason in Supplementary Table S1.

The review was restricted to health professionals, and studies of firefighters, search-and-rescue teams and mixed disaster-responder samples were excluded. This boundary was chosen because the review’s organising constructs are specific to sustained clinical caregiving, and the sensitivity analysis reported above indicates that relaxing it worsens rather than clarifies the prevalence estimate; it nonetheless limits generalisability to the wider disaster response workforce. All included studies were cross-sectional or qualitative, so no causal inference about the protective role of resilience is warranted: the observed associations are equally compatible with distress eroding self-reported resilience as with resilience buffering distress, and the two structural models, being fitted to cross-sectional data, do not resolve this. Several included reports contained internal inconsistencies between text and tables; where these could not be reconciled we treated the tables as authoritative and did not extract the disputed figures, but this necessarily introduces some uncertainty into the extracted dataset. The pooled correlation rests on four independent samples, which is few for a random-effects model, and the positive association between resilience and sleep disturbance reported in one structural model is an isolated, unreplicated finding that we report as exploratory and do not interpret mechanistically. Finally, all included studies were conducted in Türkiye following a single disaster, in a health system and cultural context with particular features—including the salience of religious coping—that may not transfer to other settings.

## Conclusion

The experience of the Kahramanmaraş earthquakes provides a sobering but instructive case study of the psychological challenges faced by healthcare workers in the wake of mass trauma. Our systematic review and meta-analysis show that the impact on these frontline providers was severe—a substantial minority screened positive for PTSD or high stress—and they also identify factors that consistently accompanied better or worse outcomes. Resilience, hardiness and adaptive coping were uniformly and inversely associated with distress, whereas exhaustion, lack of support and maladaptive coping accompanied worse outcomes; because the contributing evidence is cross-sectional, the direction of these relationships cannot be established, and they are reported as associations rather than as demonstrated causes. What can be said with more confidence is that the correlates of distress in this workforce were not confined to the individual: organisational conditions were implicated as consistently as personal resources. To support health workers after disasters, both levels therefore warrant attention—helping individuals process trauma and build coping skills, while creating work environments that prioritise their well-being. A multifaceted approach that strengthens personal resources, fosters team support and implements systemic protections is the response most consistent with the pattern of associations observed here. This both honours a duty of care to healthcare professionals and helps ensure that health systems remain capable in the face of future disasters.

Ultimately, the lessons from 2023 in Türkiye should inform a new standard in disaster response planning: one that treats the psychological welfare of health workers as an integral component of disaster readiness, on par with securing physical resources. Such an approach will build a more resilient healthcare workforce—one prepared not only to save lives in the immediate aftermath of catastrophe, but also to heal and recover in the long run.

## Data Availability

The original contributions presented in the study are included in the article/Supplementary material, further inquiries can be directed to the corresponding author.
