# Supplementary material for: Psychological impact and resilience among healthcare workers following the 2023 Kahramanmaraş earthquakes: systematic review and meta-analysis
Source: Front Psychol. 2026 Oct 7;17:1779042. doi: 10.3389/fpsyg.2026.1779042 (PMC13682973; doi:10.3389/fpsyg.2026.1779042)
Supplement: Supplementary file 1 [file Table_1.docx]

**Supplementary Table S1. Studies Assessed at Full Text and Excluded, with Reasons**

All records below were retrieved and assessed at full text. Exclusion codes: **P** = population outside eligibility (not health professionals, or no separately reported health-professional subgroup); **O** = outcome not eligible (no validated psychological or resilience/coping measure); **D** = publication date outside the search window.

| **Study** | **n** | **Population** | **Code** | **Reason** |
| --- | --- | --- | --- | --- |
| Bakirci, Sar & Cetin (2024), J Behav Health Serv Res, 51(4), 529–544 | 223 | Firefighters, Hatay | P | Non-clinical first responders; no health-professional subgroup. Excluded on the stated population criterion. Reports 36/223 (16.1%) above PCL-5 ≥41 and PCL-5 × BRS r = −0.436; both are used in the first-responder sensitivity analysis (Supplementary Table S4). |
| Özbakır (2025), Psychiatry International, 6(3), 102 | 619 | AFAD search-and-rescue personnel | P | Non-clinical first responders. Within the search window (August 2025); excluded on population, not date. |
| İşeri & Baltacı (2024), Disaster Med Public Health Prep, 18, e128 | 170 | Firefighters, SAR workers and volunteers | P | Mixed non-clinical responder sample; 35.3% (60/170) probable PTSD reported for the whole sample only, with no health-professional subgroup. Used in the first-responder sensitivity analysis (Supplementary Table S4). |
| Hugelius, Murphy & Blomberg (2024), BMC Emerg Med, 24, 226 | 525 | Local (18%) and international (81%) disaster responders | P, O | Mixed responder population; outcome is self-reported occurrence of physical or mental health problems during deployment, not a validated psychological instrument. |
| Çakaloğlu et al. (2025), Psychology, Health & Medicine | 21 | Search-and-rescue volunteers | P | Non-clinical first responders. |
| Sert, Gülbahar Eren, Koç & Yürümez (2024), Int Nurs Rev, 72, e12920 | 232 | Nurses at a hospital outside the affected region | P | Participants were not present in any affected province, were not deployed, and no clinical care of earthquake survivors is documented; the sample therefore falls outside the predefined population. Assessed at full text during revision and excluded. |
| “Beyond the Immediate Impact” (2026), Healthcare, 14(12), 1599 | 640 | HCWs, tertiary hospital in an affected province | D | Published after the 30 September 2025 cut-off. Noted in the Limitations for future updates (54.1% PTSD risk at one year). |
| Tunçez & Özteke Kozan (2026), J Health Psychology | 27 | Mental health professionals | D | Published after the search cut-off. |

Note. Studies retrieved through the first-responder terms in the expanded population block (see Supplementary Table S2) but excluded at title and abstract on the population criterion are counted in Figure 1 and are not itemised individually here; only records assessed at full text appear above.
