# Supplementary material for: Psychological impact and resilience among healthcare workers following the 2023 Kahramanmaraş earthquakes: systematic review and meta-analysis
Source: Front Psychol. 2026 Oct 7;17:1779042. doi: 10.3389/fpsyg.2026.1779042 (PMC13682973; doi:10.3389/fpsyg.2026.1779042)
Supplement: Supplementary file 2 [file Table_2.docx]

**Supplementary Table S2. Database-Specific Search Strategies**

Search period: 6 February 2023 to 30 September 2025. Updated search run September 2026. Languages: English and Turkish. No filter was applied on indexing status, document type, publication venue or sample size. The population block is deliberately broader than the review's eligibility criteria so that population-based exclusions are documented rather than implicit; records retrieved through first-responder terminology were screened against the population criterion and, where ineligible, recorded in Supplementary Table S1.

**PubMed**

```
(("Kahramanmaraş"[tiab] OR "Kahramanmaras"[tiab] OR "Pazarcik"[tiab] OR "Elbistan"[tiab]
  OR "Turkiye earthquake"[tiab] OR "Turkey earthquake"[tiab] OR earthquake*[tiab])
AND
("healthcare worker*"[tiab] OR "health care worker*"[tiab] OR "health personnel"[tiab]
  OR "medical staff"[tiab] OR nurse*[tiab] OR physician*[tiab] OR doctor*[tiab]
  OR midwife*[tiab] OR midwives[tiab] OR "allied health"[tiab] OR "mental health professional*"[tiab]
  OR "first responder*"[tiab] OR firefighter*[tiab] OR "fire fighter*"[tiab]
  OR "search and rescue"[tiab] OR "rescue worker*"[tiab] OR "disaster responder*"[tiab]
  OR "emergency medical services"[tiab] OR paramedic*[tiab] OR "emergency personnel"[tiab]
  OR AFAD[tiab])
AND
("mental health"[tiab] OR PTSD[tiab] OR "post-traumatic stress"[tiab] OR "posttraumatic stress"[tiab]
  OR depression[tiab] OR anxiety[tiab] OR "psychological distress"[tiab]
  OR "secondary traumatic stress"[tiab] OR burnout[tiab] OR "compassion fatigue"[tiab]
  OR sleep[tiab] OR resilience[tiab] OR hardiness[tiab] OR coping[tiab]
  OR "post-traumatic growth"[tiab]))
```

Filters: publication dates 2023–2025. No language filter applied at the database level; language eligibility was applied at screening.

**Scopus**

```
TITLE-ABS-KEY(kahramanmaras OR "kahramanmaraş" OR pazarcik OR elbistan
  OR "turkey earthquake" OR "turkiye earthquake" OR earthquake*)
AND TITLE-ABS-KEY("healthcare worker*" OR "health care worker*" OR "health personnel"
  OR "medical staff" OR nurse* OR physician* OR doctor* OR midwife* OR "allied health"
  OR "first responder*" OR firefighter* OR "search and rescue" OR "rescue worker*"
  OR "disaster responder*" OR "emergency medical services" OR paramedic*
  OR "emergency personnel" OR AFAD)
AND TITLE-ABS-KEY("mental health" OR PTSD OR "post-traumatic stress" OR depression
  OR anxiety OR "psychological distress" OR "secondary traumatic stress" OR burnout
  OR "compassion fatigue" OR sleep OR resilience OR hardiness OR coping
  OR "post-traumatic growth")
```

Filters: years 2023–2025. No document-type restriction (the previous restriction to Article was removed at revision).

**Web of Science (Core Collection)**

```
TS=(kahramanmaras OR "kahramanmaraş" OR pazarcik OR elbistan OR "turkey earthquake"
   OR "turkiye earthquake" OR earthquake*)
AND TS=("healthcare worker*" OR "health care worker*" OR "health personnel" OR "medical staff"
   OR nurse* OR physician* OR doctor* OR midwife* OR "allied health" OR "first responder*"
   OR firefighter* OR "search and rescue" OR "rescue worker*" OR "disaster responder*"
   OR "emergency medical services" OR paramedic* OR "emergency personnel" OR AFAD)
AND TS=("mental health" OR PTSD OR "post-traumatic stress" OR depression OR anxiety
   OR "psychological distress" OR "secondary traumatic stress" OR burnout
   OR "compassion fatigue" OR sleep OR resilience OR hardiness OR coping
   OR "post-traumatic growth")
```

Indexes: SCI-EXPANDED, SSCI, ESCI. Years 2023–2025.

**PsycINFO and CINAHL (EBSCOhost)**

```
(kahramanmaras OR pazarcik OR elbistan OR "turkey earthquake" OR "turkiye earthquake"
  OR earthquake*)
AND ("health* worker*" OR "health personnel" OR "medical staff" OR nurs* OR physician*
  OR doctor* OR midwif* OR "allied health" OR "first responder*" OR firefighter*
  OR "search and rescue" OR "rescue worker*" OR "disaster responder*" OR paramedic*
  OR "emergency personnel" OR AFAD)
AND ("mental health" OR PTSD OR "post?traumatic stress" OR depress* OR anxiet*
  OR "psychological distress" OR "secondary traumatic stress" OR burnout
  OR "compassion fatigue" OR sleep OR resilien* OR hardiness OR coping)
```

Applied with the SU (subject) and TI/AB fields; limiters: 2023–2025.

**DergiPark and ULAKBİM TR Dizin (searched separately)**

```
(Kahramanmaraş OR Kahramanmaras OR Pazarcık OR Elbistan OR deprem)
VE
(sağlık çalışanı OR sağlık personeli OR hemşire OR doktor OR hekim OR ebe
  OR paramedik OR "acil sağlık hizmetleri" OR "acil personel" OR itfaiyeci
  OR "arama kurtarma" OR "ilk müdahale ekipleri" OR "afet müdahale"
  OR "kurtarma görevlisi" OR AFAD)
VE
(ruh sağlığı OR travma OR TSSB OR "travma sonrası stres" OR psikolojik etki
  OR depresyon OR anksiyete OR dayanıklılık OR "psikolojik sağlamlık"
  OR "başa çıkma" OR "ikincil travma" OR "ikincil travmatik stres"
  OR tükenmişlik OR "merhamet yorgunluğu" OR uyku
  OR "travma sonrası büyüme")
```

Years 2023–2025. Turkish-language records.

**Google Scholar**

```
("Kahramanmaras earthquake" OR "Turkiye earthquake" OR "Turkey earthquake")
  AND (healthcare worker OR nurse OR physician OR paramedic OR "first responder")
  AND (PTSD OR depression OR anxiety OR resilience OR coping)
```

First 200 records by relevance screened, 2023–2025, patents and citations excluded.

**Additional search procedures**

– Reference lists of all included studies were screened manually (backward citation chasing).

– Forward citation searching was performed in Web of Science and Google Scholar for every included study.

– The reference list supplied by a peer reviewer during the review of this manuscript was screened as an additional identification stream and is shown separately in Figure 1.

– Duplicate records across sources were removed before screening.

– No restrictions were placed on study design, indexing status, publication venue or sample size.

– Editorials, commentaries and conference abstracts without extractable outcome data were excluded at full-text screening and are recorded in Supplementary Table S1.
