# Supplementary material for: Psychological impact and resilience among healthcare workers following the 2023 Kahramanmaraş earthquakes: systematic review and meta-analysis
Source: Front Psychol. 2026 Oct 7;17:1779042. doi: 10.3389/fpsyg.2026.1779042 (PMC13682973; doi:10.3389/fpsyg.2026.1779042)
Supplement: Supplementary file 3 [file Table_3.docx]

**Supplementary Table S3. Risk of Bias Assessment (JBI Critical Appraisal Tools)**

All twenty included studies were appraised independently by two reviewers using the JBI tool appropriate to each design. Ratings are: Y = yes, N = no, U = unclear, NA = not applicable. The transparent overall-rating rule was applied identically to studies included in the original synthesis and to those added at revision.

**Overall rating rule.** Low–Moderate: most items rated yes, with no more than one unclear or no in a key domain. Moderate: key weakness in the confounding domain, or key reporting unclear. Moderate–High: multiple key items unclear or no, particularly the exposure and outcome measurement domains together with the confounding domain.

**S3a. Analytical cross-sectional studies (JBI checklist; n = 14)**

Q1 inclusion criteria clearly defined; Q2 subjects and setting described; Q3 exposure measured validly and reliably; Q4 objective, standard criteria for measurement of the condition; Q5 confounders identified; Q6 strategies to deal with confounding stated; Q7 outcomes measured validly and reliably; Q8 appropriate statistical analysis.

| **Study** | **Q1** | **Q2** | **Q3** | **Q4** | **Q5** | **Q6** | **Q7** | **Q8** | **Overall risk of bias** |
| --- | --- | --- | --- | --- | --- | --- | --- | --- | --- |
| Aksoy et al. (2025) | U | Y | U | Y | N | N | Y | Y | **Moderate** |
| Çifçi & Kılınç (2024) | Y | Y | Y | Y | Y | Y | Y | Y | **Low–Moderate** |
| Çobanoğlu & Oğuzhan (2024) | Y | Y | Y | Y | Y | Y | Y | Y | **Low–Moderate** |
| Dağlar et al. (2025) | Y | Y | U | Y | N | N | U | Y | **Moderate–High** |
| Gökçek et al. (2024) | Y | Y | U | Y | Y | Y | Y | U | **Moderate** |
| Kınık et al. (2024) | Y | Y | Y | Y | Y | Y | Y | Y | **Low–Moderate** |
| Kıymış & Yüce (2025) | Y | Y | Y | Y | N | N | Y | Y | **Moderate** |
| Koca et al. (2025) | U | Y | Y | Y | N | N | Y | Y | **Moderate** |
| Onat et al. (2025) | Y | Y | Y | Y | Y | Y | Y | Y | **Low–Moderate** |
| Polat H. et al. (2025) | Y | Y | U | Y | N | N | Y | Y | **Moderate** |
| Polat I. et al. (2025) | Y | Y | U | Y | N | N | Y | Y | **Moderate** |
| Satılmış et al. (2024) | Y | Y | U | Y | Y | Y | Y | Y | **Low–Moderate** |
| Şehlikoğlu et al. (2024) | Y | Y | U | Y | Y | N | Y | Y | **Moderate** |
| Topkara et al. (2024) | Y | Y | U | Y | N | N | Y | Y | **Moderate** |

Notes. For Onat et al. (2025), exposure was operationalised as the primary predictor construct (coping style), assessed with a standardised measure and modelled as a predictor. For Gökçek et al. (2024), Q8 was rated unclear because the prevalence of anxiety reported in the Discussion (28.9%) differs from that given in the study’s own results table (46.5%); the tabulated value was used for extraction. For Satılmış et al. (2024), the proportion of male participants differs between the abstract (62.7%) and the results and table (55.7%); the tabulated value was used. For Şehlikoğlu et al. (2024), Q6 was rated no because group comparisons were bivariate and no multivariable adjustment was undertaken; Q3 was rated unclear because participants employed in the affected provinces before the earthquakes and those assigned afterwards were analysed together.

**S3b. Case-control study (JBI checklist; n = 1)**

C1 groups comparable other than the presence of the condition; C2 cases and controls matched appropriately; C3 same criteria used for identification of cases and controls; C4 exposure measured in a standard, valid and reliable way; C5 exposure measured in the same way for cases and controls; C6 confounding factors identified; C7 strategies to deal with confounding stated; C8 outcomes assessed in a standard, valid and reliable way; C9 exposure period of interest adequate; C10 appropriate statistical analysis.

| **Study** | **C1** | **C2** | **C3** | **C4** | **C5** | **C6** | **C7** | **C8** | **C9** | **C10** | **Overall risk of bias** |
| --- | --- | --- | --- | --- | --- | --- | --- | --- | --- | --- | --- |
| Emirza et al. (2025) | U | N | Y | Y | Y | Y | Y | Y | Y | U | **Moderate–High** |

Notes. The design is a comparative study in which the grouping variable is deployment to the earthquake zone rather than a disease state, so several items of the case-control checklist apply only loosely. C2 was rated no because cases and controls were not matched and differed significantly on gender, occupational group and prior earthquake experience. C10 was rated unclear because the reported correlations between total compassion fatigue and the coping subscales (r = .805 to .847) are not reconcilable with the correlations reported for both compassion fatigue sub-dimensions against the same subscales (r = −.135 to .157), and because the direction of the social-support finding differs between the results and the discussion. This study contributes to the narrative synthesis only and to no pooled estimate.

**S3c. Qualitative studies (JBI checklist; n = 5)**

Q1 congruity between the stated philosophical perspective and the research methodology; Q2 congruity between the methodology and the research question; Q3 congruity between the methodology and the data collection methods; Q4 congruity between the methodology and the analysis of data; Q5 congruity between the methodology and the interpretation of results; Q6 researcher located culturally or theoretically; Q7 influence of the researcher addressed; Q8 participants and their voices adequately represented; Q9 ethical approval and consent; Q10 conclusions flow from the analysis.

| **Study** | **Q1** | **Q2** | **Q3** | **Q4** | **Q5** | **Q6** | **Q7** | **Q8** | **Q9** | **Q10** | **Overall risk of bias** |
| --- | --- | --- | --- | --- | --- | --- | --- | --- | --- | --- | --- |
| Deniz Doğan et al. (2024) | Y | Y | U | Y | Y | U | U | Y | Y | Y | **Moderate** |
| Emirza et al. (2024) | Y | Y | Y | Y | Y | Y | Y | Y | Y | Y | **Low** |
| Köksal et al. (2025) | Y | Y | Y | Y | Y | Y | U | Y | Y | Y | **Low–Moderate** |
| Mert & Köksal (2025) | U | Y | Y | Y | Y | Y | Y | Y | Y | Y | **Low–Moderate** |
| Uysal et al. (2025) | Y | Y | Y | Y | Y | U | U | Y | Y | Y | **Low–Moderate** |

Notes. For Deniz Doğan et al. (2024), Q3 was rated unclear because nineteen of the thirty interviews were not audio-recorded and were captured as contemporaneous notes only; Q6 and Q7 were rated unclear because researcher positioning and reflexivity are limited, and the ethics approval was identified only in the published corrigendum. For Mert and Köksal (2025), Q1 was rated unclear because the study is described as descriptive qualitative without an explicit philosophical position; researcher positioning and reflexivity are, by contrast, reported in unusual detail.

**Summary of ratings**

| **Design** | **Low** | **Low–Moderate** | **Moderate** | **Moderate–High** |
| --- | --- | --- | --- | --- |
| Cross-sectional (n = 14) | 0 | 5 | 8 | 1 |
| Case-control (n = 1) | 0 | 0 | 0 | 1 |
| Qualitative (n = 5) | 1 | 3 | 1 | 0 |
| **Total (n = 20)** | **1** | **8** | **9** | **2** |

Neither study rated at moderate-to-high risk of bias contributes to a pooled estimate: Dağlar et al. (2025) reports a correlation between two coping subscales rather than between a resilience-related resource and a traumatic stress outcome, and Emirza et al. (2025) contributes to the narrative synthesis only. Restricting either meta-analysis to studies rated below moderate-to-high therefore reproduces the primary model exactly, as reported in Supplementary Table S4.
