# Supplementary material for: Psychological impact and resilience among healthcare workers following the 2023 Kahramanmaraş earthquakes: systematic review and meta-analysis
Source: Front Psychol. 2026 Oct 7;17:1779042. doi: 10.3389/fpsyg.2026.1779042 (PMC13682973; doi:10.3389/fpsyg.2026.1779042)
Supplement: Supplementary file 4 [file Table_4.docx]

**Supplementary Table S4. Sensitivity Analyses**

| **Analysis** | **k** | **Pooled prevalence (95% CI)** | **I²** | **τ²** |
| --- | --- | --- | --- | --- |
| Primary model (all eligible studies) | 5 | **36.3% (28.2–45.4)** | **86.2%** | **0.140** |
| Direct survivors only | 2 | **43.8% (36.1–51.9)** | **80.6%** | **0.044** |
| Deployed responders only | 2 | **19.5% (3.5–61.8)** | **92.8%** | **1.752** |
| Leave-one-out: omitting Aksoy et al. | 4 | **32.3% (23.0–43.4)** | **83.2%** | **0.176** |
| Leave-one-out: omitting Polat H. et al. | 4 | **33.7% (22.8–46.8)** | **89.1%** | **0.252** |
| Leave-one-out: omitting Polat I. et al. | 4 | **42.0% (36.9–47.3)** | **61.6%** | **0.028** |
| Leave-one-out: omitting Satılmış et al. | 4 | **35.5% (26.1–46.2)** | **89.4%** | **0.164** |
| Leave-one-out: omitting Şehlikoğlu et al. | 4 | **34.5% (24.4–46.2)** | **89.4%** | **0.197** |
| By instrument: PCL-5 ≥47 | 2 | **39.0% (33.2–45.1)** | **0.0%** | **0.000** |
| By instrument: IES-R ≥33 | 2 | **22.8% (2.9–74.6)** | **95.9%** | **2.629** |
| Excluding studies rated moderate–high risk of bias | 5 | **36.3% (28.2–45.4)** | **86.2%** | **0.140** |
| First-responder sensitivity (+Bakirci 2024; +İşeri & Baltacı 2024) | 7 | **31.5% (23.2–41.3)** | **92.4%** | **0.282** |
| Non-clinical first responders only | 2 | **24.5% (10.5–47.4)** | **94.6%** | **0.513** |

Note. Random-effects models, DerSimonian–Laird estimator, logit-transformed proportions, computed in R 4.3.1 (**meta**, **metafor**). All twenty included studies were appraised (Supplementary Table S3). Neither of the two studies rated moderate-to-high risk of bias contributes to a pooled estimate, so the risk-of-bias-restricted models reproduce the primary models exactly. Restricting further to studies rated low or low-to-moderate was not undertaken, since it would leave one study in the prevalence model and two in the correlation model.

**Correlation synthesis (resilience-related measures and traumatic stress)**

| **Analysis** | **k** | **Pooled r (95% CI)** | **I²** |
| --- | --- | --- | --- |
| Primary model (one effect per independent sample) | 4 | **−0.420 (−0.522 to −0.307)** | **79.1%** |
| First-responder sensitivity (+Bakirci 2024, PCL-5 × BRS) | 5 | **−0.423 (−0.504 to −0.335)** | **72.3%** |
| Excluding studies rated moderate–high risk of bias | 4 | **−0.420 (−0.522 to −0.307)** | **79.1%** |

Note. Fisher's z-transformed Pearson coefficients, DerSimonian–Laird random-effects model, back-transformed for presentation. The protective association is essentially unchanged by the population boundary.
